# The role of zinc on nutritional status, sarcopenia, and frailty in older adults: a scoping review

**DOI:** 10.1093/nutrit/nuad094

**Published:** 2023-08-07

**Authors:** Hansani Madushika Abeywickrama, Mieko Uchiyama, Tomoko Sumiyoshi, Akiko Okuda, Yu Koyama

**Affiliations:** Department of Nursing, Graduate School of Health Sciences, Niigata University, Niigata, Japan; Department of Nursing, Graduate School of Health Sciences, Niigata University, Niigata, Japan; Department of Nursing, Graduate School of Health Sciences, Niigata University, Niigata, Japan; Department of Medical Technology, Graduate School of Health Sciences, Niigata University, Niigata, Japan; Department of Nursing, Graduate School of Health Sciences, Niigata University, Niigata, Japan

**Keywords:** Frailty, older adults, nutritional status, sarcopenia, scoping review, zinc

## Abstract

**Background:**

Zinc (Zn) deficiency, malnutrition, sarcopenia, and frailty are prevalent among older adults and are prominent factors contributing to disability and mortality.

**Objective:**

This scoping review was conducted to aid understanding of the extent and types of research addressing the role of Zn in nutritional status, sarcopenia, and frailty, among older individuals.

**Method:**

A systematic search was performed in August 2022 of 3 electronic databases (PubMed, Web of Science, and ProQuest) using predefined search terms. The review was conducted referring to the Arksey and O’Malley framework and PRISMA-ScR.

**Results:**

The search retrieved 16 018 records, and a total of 49 studies were included in this review after the screening. Of those, 30 were based on dietary Zn intake, 18 on tissue Zn levels, and 1 on both. Most studies were based on cross-sectional data from community-dwelling older adults. Studies addressing the associations between Zn status and individual anthropometric and sarcopenia-related variables reported inconsistent results. However, most studies reported inverse associations between malnutrition, frailty, and Zn status.

**Conclusion:**

There was more consistent evidence of the relationship of Zn status with malnutrition, sarcopenia, and frailty rather than with individual nutritional parameters. Validated screening and assessment tools and criteria and prospective studies are required to elucidate the relationship of Zn with sarcopenia and frailty in the older population.

## INTRODUCTION

With the accelerated pace of population aging globally, sarcopenia and frailty have become leading health problems among older adults. The World Health Organization (WHO) defines the concept of healthy aging as the “development and maintenance of the functional ability to enable well-being in older age.”^1^ Both sarcopenia and frailty are characterized by loss of muscle mass (MM) and function, making individuals susceptible to functional disability^2^ and reducing the opportunity to experience healthy aging.

Increasing age is associated with changes in body composition, such as an increase in fat mass and abdominal fat accumulation and a decrease in fat-free mass. Sarcopenia is a muscle disorder commonly found in the geriatric population and characterized by low MM, low muscle strength, and/or decreased physical function.^3^ Frailty is a common geriatric syndrome caused by the multisystemic reduction of physiological capacities, which increases vulnerability to stress. Nutritional frailty refers to a sudden significant weight loss together with loss of MM and strength that makes older adults susceptible to disability.^4^ The etiology of frailty is multifaceted and includes nutritional problems such as malnutrition and obesity. Recent studies have revealed the simultaneous presence of frailty and obesity among increasing numbers of older adults.^5^

The nutrient intake and nutritional status of older people are affected by several factors associated with increased age. First, age-associated physiologic changes, such as the reduced capacity to absorb and use nutrients, put older adults at risk of macro and micronutrient deficiencies. Second, older adults are required to consume micronutrients in greater densities due to lower energy requirements. Third, many older adults are suffering from chronic diseases that may be associated with their diet, making provision of optimal nutrition and maintaining adequate stores of essential vitamins and minerals problematic. Last, alterations in appetite, taste, and swallowing, resulting from the intrinsic changes associated with the aging process, the disease itself, and/or treatments a person is receiving, can affect the eating habits of elderly individuals.^6^^,^^7^ The decrease in appetite and/or food intake with aging, referred to as “anorexia of aging,” increases the risk of protein-energy malnutrition, sarcopenia, and frailty in older people.^8^

Zinc (Zn) is an important nutrient that influences the nutritional intake and nutritional status of older people because it affects taste and appetite. Older adults are susceptible to Zn deficiency due to many factors, including lower consumption of Zn-rich foods, which can be caused by the decline in taste acuity, poor dentition, inadequate mastication of food; and also intake of foods that reduce the bioavailability of Zn, or the presence of non-communicable diseases.^9^ Zn deficiency is potentially associated with impaired taste acuity, because salivary Zn is linked with gustatory nerve activity.^10^ Particularly, a decline in salt taste acuity with advancing age is associated with Zn status.^11^ Zn deficiency can result in reduced appetite through interaction with leptin,^12^ leading to or resulting from reduced intake of foods high in Zn by older adults. As such, Zn deficiency is both a cause and an effect of the loss of taste acuity with aging. Along with the poor appetite and dysphagia common among older people, impaired taste perception reduces the pleasure of eating and contributes to malnutrition.

In light of these facts, in this scoping review, we aimed to (1) identify the scope and nature of research related to the role of Zn and nutritional health of older adults; (2) examine potential relationships among Zn deficiency, nutritional status, sarcopenia, and frailty among older adults; and (3) identify gaps in the existing literature and research priorities that will better inform evidence-based practice and policies.

## MATERIALS AND METHODS

This scoping review was conducted and reported referring to Arskey and O’Malley’s 6-stage methodological framework (excluding the last and optional stage of consulting with stakeholders)^13^ and the recommendations made by Levac et al^14^ and Preferred Reporting Items for Systematic Reviews and Meta-Analyses Statement for Reporting Scoping Reviews (PRISMA-ScR; see Table S1 in the Supporting Information online).^15^

### Stage 1: Develop the research question

Levac et al^14^ recommended developing the research question by clearly clarifying the concept, population, and outcomes of interest to establish the focus of the review and an effective search strategy. As such, the concept of this scoping review was defined as a type of review that aims to identify the key concepts, theories, sources of evidence, and research gaps by collating the available literature on a particular topic.

This review was guided by the broad question: what kind of evidence is available in the existing literature about the potential associations between nutritional status, sarcopenia, frailty, and Zn in older adults? Three specific questions were to be answered by the review. (1) Is there a relationship between Zn status and nutritional parameters? (2) Does such relationships influenced by the method of nutritional screening or evaluation of Zn status? (3) What are the existing research gaps?

The study population was clarified as older adults (≥60 years; or specific definitions for older people). Health outcomes of interest were the relationship of Zn status with nutritional status, physical performance, sarcopenia, and frailty. Zn status, which was assessed in terms of dietary intake or concentrations in serum, hair, or nails, was considered. The nutritional status, which measured as body mass index (BMI), body composition (body fat % and body muscle %), body circumferences (hip, waist, calf, mid-upper arm, and middle arm), or physical fitness, and nutritional risk, sarcopenia, or frailty, screened using any definition, were included in the review.

### Stage 2: Identify relevant studies

A comprehensive literature search was conducted in 3 electronic databases: PubMed (US National Library of Medicine), Web of Science (Thomson Reuters), and ProQuest (Clarivate) in August 2022. No filters or limits were applied to widen the search. The search strategy used for PubMed is provided in File S1 in the Supporting Information online. The references of the extracted studies and relevant reviews, and suggested articles by PubMed were used to identify additional studies. Records retrieved from database searches were imported into Endnote software, version 20.2 (Clarivate). The software was used to remove the duplicates.

### Stage 3: Screening and study selection

The preliminary screening of the titles and abstracts of extracted reports was conducted by 1 reviewer (H.M.A.). Secondary review of titles, abstracts, and article content for inclusion was done by another reviewer (Y.K.) independently. After consensus was reached, the full text of the selected studies was reviewed by the first reviewer to confirm adherence to eligibility criteria. The population, interventions, comparators, outcomes, and study design (PICOS) criteria were applied to include and exclude studies during the screening process (Table 1).

**Table 1:** PICOS criteria for inclusion of studies

PICOS parameter	Inclusion criteria	Exclusion criteria
Population	Studies that included older participants (aged ≥60 y, or else applicable definitions used in the selected studies) Community-dwelling older adults, adults who reside in nursing homes, outpatients of geriatric clinics, or inpatients in acute care wards	Children, adolescent, and adult study populations (aged <60 y or did not define as older adults by applicable definitions used in the selected studies) Older adults with life-threatening conditions or systemic diseases such as cancer, cardiac diseases, chronic obstructive pulmonary disease, muscle disorders, chronic kidney disease, neurological conditions, or who were admitted to an intensive care unit or other similar care.
Interventions	Studies that quantitatively assessed intake or serum and tissue concentrations of Zn, and assessed nutritional status, sarcopenia, or frailty	Qualitative studies
Comparators	Older adults with and without Zn deficiency (inadequate Zn intake or lower serum or tissue Zn concentrations) Older adults with normal nutritional status (body mass index, anthropometric and body composition measures) or physical function (Short Physical Performance Battery score, hand-grip strength, gait speed) compared with those with below and/or above normal conditions Older adults with and without sarcopenia or frailty	
Outcomes	Studies with clearly reported the findings related to the review question (Zn intake or Zn status related to nutritional status, sarcopenia, or frailty)	Duplicate publications from the same studies Articles written in languages other than English
Study design	All study designs	Non–data-driven articles such as editorials, commentaries, letters to the editor, expert opinion, case reports, reviews Animal studies

### Stage 4: Extract data

The following information was extracted from the articles selected after the full-text screening: author(s); year of publication; study location; methodology (design, living arrangement, inclusion and exclusion criteria); sample characteristics (sample size, age, sex, related nutritional data); assessment method of Zn (Zn intake or Zn levels in serum, hair, or nails); variables related to nutritional status, sarcopenia, or frailty; the definitions used to screen nutritional status, sarcopenia, or frailty; and findings related to the review question. Two reviewers (H.M.A. and Y.K.) independently extracted and then reviewed the data with the other authors. The data extracted are summarized^16–46^ in Tables 2 and 3.^47–64^

**Table 2:** Characteristics and main findings of studies that reported relationships of Zn intake with nutritional status, sarcopenia, and frailty

Reference	Study area	Study design and sample	Assessment of Zn intake and nutritional status	Primary findings related to the review question
Al-Majali et al (2021)^16^	Jordan	Sex-matched, case-control study Study sample: Adults aged >60 y and living in elderly care centers or visited private clinics in geriatric medicine N = 50 (25 with sarcopenia and 25 without)	1. Zn intake by 1-d 24-h dietary recall 2. Sarcopenia (criteria not clearly defined)	1. Zn intake was not associated with sarcopenia. The mean (SD) Zn intake in the 2 groups was as follows: Sarcopenia: 6.19 (2.5); no sarcopenia: 6.1 (2.7); *P* = 0.946 2. Zn intake in the sarcopenic group was significantly low (*P* ≤ 0.001) compared with the recommendation (mean ± SD) (9.44 ± 1.5).
Aparicio-Ugarriza et al (2019)^17^	Madrid and Mallorca, Spain	A cross-sectional, multicenter study Study sample: Adults aged >55 y N = 324	1. Zn intake by 2 nonconsecutive 24-h dietary recalls 2. PF^a^ by CST, 8-foot TUG test, 6-min walk test, and HGS	1. Zn intake was not associated with PF. β = 0.189234; 95%CI, −0.313060, 0.691527; *P* = 0.459 2. Zn intake among men with medium and high levels of PF was higher than in women (*P* < 0.01). Median (5th–95th percentiles) Zn intake in PF levels^a^: Low PF: M, 9.6 (5.0–17.7); F, 8.9 (3.7–14.4) Medium PF: M, 9.4 (5.7–19.9), F, 8.0 (4.3–17.1) High PF: M, 10.5 (6.0–16.1); F, 8.3 (4.4–20.5) 3. Prevalence of low Zn intake (<EAR) was comparatively low among women in each PF level^a^: Low PF: M, 50%; F, 30% Medium PF: M, 52%; F, 35% High PF: M, 47%; F, 30%
Asamane et al (2020)^18^	Birmingham, West Midland, United Kingdom	Observational, longitudinal study (baseline and 8-mo follow-up) Study sample: community-dwelling, ethnically diverse adults (≥60 y) Sample size: At baseline: n = 100; at follow-up: n = 81	1. Zn intake by 2 nonconsecutive 24-h recalls 2. Nutritional status by HGS, SPPB (balance test, 4-m GS, 5-times CST)	Zn intake was not associated with SPPB and HGS at baseline or follow-up.
Carrier et al (2021)^19^	4 provinces in Canada: Alberta, Manitoba, New Brunswick, Ontario	Cross-sectional study Study sample: Residents >65 y old in long-term-care homes N = 619	1. Zn intake by weighed food intake on 3 nonconsecutive days 2. Nutritional status by CC	No significant association between Zn intake and low CC (<31 cm) was observed. OR (95%CI) of the logistic regression model (adjusted for home, unit, age, sex) was 0.92 (0.81–1.04).
Das et al (2020)^20^	New South Wales, Australia	Prospective study (3-y follow-up) Study sample: community-dwelling older men ≥75 y old Sample size: At baseline n = 794; at follow-up, n = 341	1. Zn intake by 3-mo diet history questionnaire 2. Frailty by Fried frailty phenotype criteria^a^	No associations were observed between the Zn intake at baseline and incident prefrailty and frailty during the 3 y. OR (95%CI) and *P* values of the fully adjusted model: Q1^a^: prefrail: 1.34 (0.72–2.51), 0.35; frail: 1.00 (0.42–2.34), 0.99 Q2^a^: prefrail: 1.39 (0.77–2.35), 0.28; frail: 0.49 (0.20–1.22), 0.13 Q3^a^: prefrail: 1.01 (0.56–1.83), 0.98; frail: 1.16 (0.51–2.65), 0.72 Q4^a^: reference *P* for trend: prefrail, *P =* 0.44; frail, *P* = 0.23
Das et al (2021)^21^	New South Wales, Australia	Cross-sectional study Study sample: community-dwelling men ≥75 y old Sample size: Findings are based on sample size of 692.	1. Zn intake by 3-mo diet history questionnaire 2. Sarcopenia^a^ determined by EWGSOP criteria, EWGSOP2 criteria, FNIH criteria	1. Zn intake and proportion of participants (%) meeting the recommended Zn intake were significantly different among older adults with and without sarcopenia according to FNIH and EWGSOP2 criteria, but not according to EWGSOP criteria. Median (IQR) Zn intake and proportion meeting the NRV (%) in sarcopenic groups were as follows: *by FNIH:* Sarcopenia: 11.9 (5.6), 50; no sarcopenia: 13.4 (5.5), 68.2; *P* for Zn intake, *P* = 0.02; NRV, *P* = 0.001 *by EWGSOP:* Sarcopenia: 13.43 (6.59), 63.4; no sarcopenia: 13.45 (5.46), 68.1; for Zn intake, *P* = 0.78; NRV, *P* = 0.4 *by EWGSOP2:* Sarcopenia: 13.03 (5.15), 64.4; probable sarcopenia: 13.28 (5.73), 68.8; severe sarcopenia: 12.95 (5.71), 60.4; no sarcopenia: 14.76 (5.36), 83.1; for Zn intake, *P* = 0.02; NRV, *P* = 0.007 2. A significant association between Zn intake and FNIH-defined sarcopenia was observed in the unadjusted model. However, significance was lost in the multivariate model. OR (95%CI) and *P* values for models were as follows: *Unadjusted model* Q4^a^: reference; Q3^a^: 0.94 (0.46–1.90), 0.86 Q2^a^: 1.17 (0.60–2.31), 0.65; Q1^a^: 1.27 (0.71–2.26), 0.43; *P* for trend = 0.03 *Adjusted model:* Q4^a^: reference; Q3^a^: 0.58 (0.26–1.31), 0.19; Q2^a^: 0.66 (0.29–1.48), 0.66; Q1^a^: 0.79 (0.33–1.85), 0.58; *P* for trend = 0.38
De Nucci et al (2022)^22^	Southern Italy	Cross-sectional study Study sample: community-dwelling adults ≥65 y old N = 1502	1. Zn intake by FFQ 2. Frailty assessed by Fried frailty phenotype criteria^a^	Zn intake was associated with frailty. Mean (SD) Zn intake in the frail 52.17 (32.14) group was significantly lower than in nonfrail group 59.43 (34.20). Effect size (95%CI) was 0.21 (0.06–0.36). By logistic regression model: OR (95%CI): 0.977 (0.952–0.998); *P* = 0.048
Ebrahimi-Mousavi et al (2022)^23^	Tehran, Iran	Cross-sectional study Study sample: community-dwelling adults ≥55 y old N = 300	1. Zn intake by FFQ 2. Sarcopenia assessed by EWGSOP criteria^a^	Zn intake was not associated with sarcopenia. Mean (SD) Zn intake in participants with sarcopenia was 12.28 (6.44); without sarcopenia: 12.30 (4.40); *P* = 0.97
Hayashi et al (2021)^24^	Kitakyushu, Japan	Cross-sectional study Study sample: Community-dwelling women ≥65 y old N = 120	1. Zn intake by FFQ 2. Frailty by J-CHS criteria^a^	Zn intake was not associated with frailty. Median (IQR) Zn intake in 2 frailty groups was as follows: prefrail: 8.5 (7.9–9.1); nonfrail: 8.5 (7.8–9.1); *P* = 0.665 No. (%) of older women with inadequate Zn intake^a^ in 2 frailty groups: prefrail, 7 (13); nonfrail, 2 (3); *P* = 0.766
Jun et al (2020)^25^	United States	Cross-sectional study Data from National Health and Nutrition Examination survey in 2011–2012 and 2013–2014 cycles Study sample: Adults ≥60 y old N = 2969	1. Zn intake by 2 24-h dietary recalls 2. Nutritional status by BMI	Zn intake was not associated with weight. Prevalence of Zn intake less than EAR by sex and weight status^a^: From food sources alone, % (SE): M: HW (n = 378), 23.5 (4.2); OW (n = 602), 20.3 (4.7); obese (n = 482), 27.2 (4.0) F: HW (n = 399), 17.4 (3.9); OW (n = 453), 21.1 (4.9); obese (n = 655), 26.6 (3.4) From total intakes, % (SE): M: HW, 16.4 (3.2); OW, 14.4 (3.5); obese, 18.5 (3.7) F: HW, 11 (2.7); OW, 12 (3.0); obese, 19.6 (2.7)
Jyvaäkorpi et al (2016)^26^	Finland	Cross-sectional study Study sample: Both home-dwelling and institutionalized people ≥60 y old without and with disabilities N = 900	1. Zn intake by 1–3-d food diaries 2. Nutritional status by MNA	Zn intake was associated with malnutrition and the risk of malnutrition. Mean (SD) Zn intake of each MNA class^a^: Malnutrition (n = 72): 8.7 (2.9) At risk (n = 449): 10 (3.3) Normal (n = 379): 10.9 (3.1); *P* < 0.001
Kaimoto et al (2021)^27^	Kagoshima, Japan	Cross-sectional study Study sample: community-dwelling adults ≥65 y old N = 815	1. Zn intake by diet history questionnaire 2. Frailty by Fried frailty phenotype criteria^a^	Zn intake was associated with frailty in women but not in men. Mean (SD) Zn intakes were, for older women: Robust (n = 237), 8.91 (1.13); prefrailty (n = 278): 8.70 (1.19); *P* = 0.033 For older men: Robust (n = 146), 9.42 (1.45); prefrailty (n = 154), 9.56 (1.58); *P* = 0.422
Keser et al (2021)^28^	Zagreb, Croatia	Cross-sectional study Study sample: Nursing home residents >65 y old N = 84	1. Zn intake by 24-h dietary recall 2. Body composition by BIA (MM, FM, and total bone mass)	Zn intake was not associated with body composition. Mean (SD) Zn intake of each group: Adiposity only^a^ (n = 22): 5.5 (2.7) Osteosarcopenic adiposity^a^ (n = 45): 5.9 (2.9); *P *>* *0.05
Khanal et al (2021)^29^	United Kingdom	Cross-sectional study Study sample: Women >60 y old N = 281	1. Zn intake by FFQ 2. Nutritional status by SMM	Zn intake was not associated with MM. Mean (SD) Zn intake by SMM_r_ category^a^: Low SMM_r_, 9.57 (3.44); high SMM_r_, 9.43 (3.26); *P* = 0.859
Kosaka et al (2013)^30^	Japan	Cross-sectional study Study sample: Disabled elderly patients residing at nursing facilities N = 26	1. Zn intake by 1-mo nutrient intake 2. Nutritional status by BMI	Zn intake was not associated with BMI. Mean Zn intake (n = 20) was not significantly different between older adults with low and normal BMI (78 mg and 92 mg, respectively; *P* > 0.05).
Ledikwe et al (2003)^31^	Pennsylvania, United States	Cross-sectional study Study sample: community-dwelling adults >65 y old N = 179	1. Zn intake by 5 random 24-h dietary recalls within 2 mo 2. Nutritional status by BMI and WC	Zn intake was associated with BMI and WC in older women but not in men. Correlation between BMI and Zn intake: F: *r* = −0.23; *P* < 0.05 M: *r* = –0.07; *P* > 0.05 Correlation between WC and Zn intake is similar to BMI.
Lengele et al (2020)^32^	Liège, Belgium	Prospective cohort study (3-y follow-up) Study sample: community-dwelling adults >65 y old N = 238	1. Zn intake by FFQ at baseline and follow-up 2. Nutritional status by 4-m GS and HGS	Zn intake was not associated with HGS or GS at baseline or 3-y follow-up. Baseline associations by linear regression: GS: β = 0.006, *P* = 0.585 HGS: β = 0.119, *P* = 0.685 Associations of longitudinal changes in Zn intake with longitudinal changes in GS and HGS: GS: β = 0.003, *P* = 0.789 HGS: β = 0.274, *P* = 0.232
Moradell et al (2021)^33^	Zaragoza, Spain	Cross-sectional study Study sample: community-dwelling adults >65 y old N = 101	1. Zn intake by FFQ 2. Frailty by SPPB score^a^ (balance, 4-m GS, CST)	Zn intake was not associated with SPPB. Mean (SD) Zn intake in each group: Robust (n = 13): 14 (0.6); prefrail (n = 68): 13 (0.3); frail (n = 20), 13.2 (0.5); *P* = 0.389
Ongan et al (2015)^34^	Turkey	Cross-sectional study Study sample: institutionalized adults >65 y old N = 554	1. Zn intake by 1-d 24-hr dietary recall 2. Nutritional status by MNA^a^	Zn intake was associated with malnutrition and risk of malnutrition. Mean (SD) intake in each group: normal (n = 245): 9.4 (3.6); at risk (n = 272): 8.5 (3.3); malnourished (n = 37): 7.1 (2.6); *P* < 0.001
Otsuka et al (2021)^35^	Japan	Cross-sectional study Study sample: community-dwelling adults ≥60 y N = 1345	1. Zn intake by diet-history questionnaire 2. Sarcopenia by AWGS criteria^a^	Zn intake was not associated with sarcopenia. Mean (SD) intakes of the 2 groups: sarcopenia: 8.1 (2.7); no sarcopenia: 8.4 (2.8); *P* = 0.400
Park et al (2022)^36^	South Korea	Cross-sectional study Study sample: Older adults aged 70–84 y from communities N = 801	1. Zn intake by 24-h recall 2. Sarcopenia by AWGS criteria^a^	Zn intake was associated with sarcopenia. Zn intake between the two groups, by least squares mean (SE): No sarcopenia: 7.22 (0.14); sarcopenia: 6.47 (0.37); *P* = 0.0583 OR (95%CI) for each Zn quartile in the multi-adjusted model: Q1: median, 3.9, n = 199; sarcopenia, 33 (reference) Q2: median, 5.7, n = 201; sarcopenia, 30: 0.92 (0.51–1.67) Q3: median, 7.3, n = 201; sarcopenia, 29): 0.78 (0.42–1.4) Q4: median, 10.2, n = 200, sarcopenia. 19): 0.39 (0.19–0.80) *P* for trend = 0.0074 Zn intake (%) according to the 2020 Dietary Reference Intakes for Koreans: No sarcopenia, 91.1 (1.9); sarcopenia, 80.0 (3.2); *P* = 0.003
Scott et al (2010)^37^	Tasmania, Australia	Prospective study Follow-up in 2–3 y (2.6 ± 0.4) Study sample: Community-dwelling people aged 50–79 y N = 740	1. Zn intake by FFQ 2. Nutritional status determined by: ALM by DEXA Muscle strength of the dominant leg during a seated isometric contraction of the knee extensors	Zn intake was associated with ALM at the baseline and ALM changes overtime. Association between ALM and energy-adjusted Zn intake at baseline: β = 0.06, *P* = 0.006 Adjusted mean values (SE) of ALM across quartiles of Zn intake: Q1: 24.36 (0.22), Q2: 24.20 (0.22), Q3: 24.68 (0.22), Q4: 25.06 (0.22); *P* for trend = 0.03 Association between change in ALM over 2.6 y and energy-adjusted Zn intake at baseline: β = 0.08, partial *R*^2^ = 0.7%, model *R*^2^ = 27%; *P* = 0.02 No association with muscle strength was observed.
Shalini et al (2020)^38^	Hyderabad City, India	Cross-sectional study Study sample: Community-dwelling people ≥60 y N = 163 Dietary data were available only for 88 participants.	1. Zn intake by 3 nonconsecutive 24-h dietary recalls (2 weekdays and 1 weekend day) 2. Frailty assessed by Fried frailty phenotype criteria^a^	Zn intake was associated with frailty. Median (IQR) Zn intake of each group: Nonfrail (n = 68): 7.2 (6.2–8.2); frail (n = 20): 5.4 (4.7–6.7); *P* < 0.001 Proportion (%) of frail older adults in Zn intake categories: <6.13: 50.6 6.13–7.82: 13.9 >7.82: 5.6 *P* < 0.001 (adjusted for age, sex, and energy)
Tamaki et al (2018)^39^	Hyogo, Japan	Cross-sectional study Study sample: Community-dwelling older people ≥65 y old N = 800 J-CHS data were available for 796 people.	1. Zn intake by 1-mo diet history questionnaire 2. Frailty assessed by KCL criteria^a^ and J-CHS criteria^a^	1. Zn intake was associated with frailty (assessed by KCL criteria) among men. Mean (SD) intake: Robust: M (n = 163), 9.98 (3.39); F (n = 313), 9.56 (3.07) Prefrail: M (n = 72), 9.88 (3.43); F (n = 185), 9.90 (3.08) Frail: M (n = 19), 7.53 (2.77); F (n = 48), 9.58 (3.55) For robust M and frail M, *P* = 0.008; for prefrail M and frail M, *P* = 0.019, *P* >0.05 (F) 2. Zn intake was not associated with frailty by J-CHS criteria. Mean (SD) intake: Robust: M (n = 104), 10.29 (3.36); F (n = 227), 9.78 (3.11); prefrail: M (n = 139), 9.46 (3.44); F (n = 294), 9.61 (3.16); frail: M (n = 9), 8.53 (2.68); F (n = 23), 9.56 (3.07) For M and F, *P* > 0.05
Tay et al (2021)^40^	New Zealand	Cross-sectional study Study sample: Community-dwelling prefrail older adults aged ≥75 y (60 y for Māori and Pacific people) N = 465	1. Zn intake by 24-h dietary recalls on 2 nonconsecutive days 2. Frailty by modified Fried frailty phenotype criteria^a^	Prevalence of inadequate Zn intake (according to EAR) in prefrail older adults: M, 82%; F, 45%
ter Borg et al (2016)^41^	Maastricht, Netherlands	Cross-sectional study Study sample: Adults ≥65 y old living at home or in an assisted or residential living facility N = 227	1. Zn intake by FFQ 2. Sarcopenia by EWGS criteria^a^	Zn intake was not associated with sarcopenia. Zn intake (without supplement intake) in the 2 groups (mean, SD): No sarcopenia (n = 173), 10 (3); sarcopenia (n = 53), 9 (3); *P* = 0.094 Zn intake (with supplement intake) in the 2 groups (mean, SD): No sarcopenia (n = 167), 12 (5); sarcopenia (n = 53), 11(6); *P* = 0.576
Vega-Cabello et al (2022)^42^	Spain	Prospective cohort study 3 follow-up waves (dietary history at baseline and first follow-up; LEF and frailty at first and third follow-up) Study sample: noninstitutionalized adults ≥60 y N = 2963	1. Zn intake by dietary history 2. LEF by SPPB score^a^ (GS, CST, standing balance) 3. Frailty assessed by modified Fried frailty criteria^a^	1. Zn intake was associated with impaired LEF (*P* = 0.03). HR (95%CI) for the associations between tertiles of Zn intake and impaired LEF: T1, reference; T2, 0.81 (0.65–1.01); T3, 0.75 (0.58–0.97); per 1-SD increment: 0.86 (0.76–0.97) 2. Zn intake was associated with frailty (*P* = 0.02). HR (95%CI) for the associations between tertiles of Zn intake and frailty: T1, reference; T2, 0.69 (0.50–0.95); T3, 0.63 (0.44–0.92) Per 1-SD increment: 0.81 (0.68–0.97) 3. Among the components of SPPB, Zn status was associated with only impaired standing balance. Fully adjusted HR per 1-SD increment (95%CI): Slow GS (≥5.7): 1.01 (0.95–1.07), *P* > 0.05 Inability to rise from a chair: 0.99 (0.93–1.05), *P* > 0.05 Impaired standing balance: 0.90 (0.81–0.99), *P* < 0.05 4. Zn intake was not associated with any individual component of the frailty criteria. Fully adjusted HR per 1-SD increment (95%CI): Weight loss: 0.98 (0.85–1.13) Exhaustion: 0.88 (0.77–1.01) Low PA: 0.99 (0.87–1.10) Slow GS: 0.90 (0.80–1.01) Low HGS: 0.94 (0.87–1.03) *P* for all components >0.05 5. Highest Zn intake and bioavailability had a lower risk of impaired LEF and frailty. 6. High Zn intake and low intake of high-phytate foods associated with a decreasing risk of impaired LEF and frailty 7. Adherence to RDA of Zn intake was associated with risk frailty in women. HR (95%CI): M: low LEF, 0.67 (0.38–1.18); frailty, 0.80 (0.34–1.89) F: low LEF, 0.89 (0.65–1.23); frailty, 0.65 (0.43–0.97)
Verlan et al (2017)^43^	United Kingdom	1:1 Age- (–1 y, +2y) and sex-matched case-control study Study sample: community-dwelling adults ≥65 y N = 66 case patients (sarcopenic) and 66 control participants (nonsarcopenic)^a^	1. Zn intake by 3-day prospective diet records (2 weekdays and 1 weekend day) 2. Sarcopenia diagnosis by specified criteria based on SPPB score (GS, CST, balance), SMI, and BMI^a^	Zn intake was not associated with sarcopenia. Mean (SD) intakes in the 2 groups were sarcopenia, −8.2 (3.0); no sarcopenia, −9.0 (2.6); *P* = 0.087
Waters et al (2014)^44^	Albuquerque, New Mexico, United States	Cross-sectional, secondary data analysis (from 1994 data) Study sample: community-dwelling older adults ≥60 y N = 315	1. Zn intake by 3-d diet records (3 successive weekdays) 2. Nutritional status by 24-foot GS	Zn intake was associated with GS. Associations (OR; 95%CI) between slow GS and Zn intake by logistic regression: M (age-adjusted), : 3.57 (1.14–11.18), *P* < 0.05 F (age and mental status adjusted), 2.33 (1.12–4.85), *P* <0.05 Mean Zn intake by sex and GS: M: slow GS (<0.8), 10.8; normal GS, 11.8 (*P* = 0.06) F: slow GS (<0.7), 8.2; normal GS, 9.0 (*P* = 0.09)
Wu et al (2022)^45^	Taiwan	Cross-sectional secondary analysis of data from a national survey from 2014–2017 Study sample: Noninstitutionalized older people ≥65 y old N = 1920 Both dietary and frailty data were available for 1186 participants.	1. Zn intake by 24-h dietary recall 2. Frailty by modified Fried frailty phenotype criteria^a^	Zn intake was associated with frailty. Age-adjusted mean (SEM) Zn intake by frailty groups: M 65–74 y old: robust, 13.33 (0.47), prefrail, 12.55 (0.64), frail, 9.63 (1); *P* (adjusted by age) = 0.001 *P* (adjusted by age and energy intake) = 0.315 M >74 y: robust, 14.91 (1.23), prefrail, 11.4 (0.63), frail, 10.82 (1.41); *P* (adjusted by age) = 0.036, *P* (adjusted by age and energy intake) = 0.015 F 65–74 y old: robust, 11.17 (0.49), prefrail, 10.56 (0.52), frail, 8 (1.24); *P* (adjusted by age) = 0.020, *P* (adjusted by age and energy intake) = 0.133 F >74 y: robust, 10.41 (0.92), prefrail, 8.09 (0.46), frail, 7.96 (0.76); *P* (adjusted by age) = 0.028, *P* (adjusted by age and energy intake) = 0.199
Yeung et al (2021)^46^	Netherlands	Cross-sectional study Study sample: Community-dwelling older adults referred to an outpatient clinic N = 58	1. Zn intake by 1-d food diary (2 weekdays and 1 weekend day) 2. Nutritional status: SMM by BIA; HGS; CST	Energy-adjusted Zn intake was not associated with any of the nutritional status parameters. 1. zSMM^b^^,c^: β = 0.00, SE = 0.04, *P* = 0.944 2. zSMI^a^^,b,c^: β = −0.03, SE = 0.04, *P* = 0.529 3. zSMM/BMI^b^ (age-adjusted): β = 0.01, SE = 0.05, *P* = 0.850 4. zHGS^b^^,c^: β = 0.02, SE = 0.06, *P* = 0.749 5. zCST^b^^,c^: β = −0.009, SE = 0.05, *P* = 0.060

a Detailed definitions are available in Table S2 in the Supporting Information online.

b Sex-specific *z* scores (standardized).

c Adjusted by age and body weight.

*Abbreviations:* ALM, appendicular lean mass; AWGS, Asian Working Group for sarcopenia; BIA, bioelectrical impedance analysis; BMI, body mass index; CC, calf circumference; CI, confidence interval; CST, chair stand test; DEXA, dual-energy X-ray absorptiometry; EAR, estimated average requirement; EWGSOP, European Working Group on Sarcopenia in Older People; F, female; FFQ, food frequency questionnaire; FM, fat mass; FNIH, Foundation of the National Health Institutes of Health; GS, gait speed; HGS, hand-grip strength; HR, hazard ratio; HW, healthy weight; IQR, inter-quartile range; J-CHS, Japanese version of the Cardiovascular Health Study; KCL, Kihon checklist; LEF, lower extremity function; M, male; MM, muscle mass; MNA, mini nutritional assessment; NRV, nutrient reference values; OR, odds ratio; OW, overweight; PA, physical activity; PF, physical fitness; Q, quartile; RDA, recommended dietary allowance; SD, standard deviation; SE, standard error; SEM, standard error of the mean; SMI, skeletal muscle index; SMM, skeletal muscle mass; SMM_r_, relative skeletal muscle mass; SPPB, short physical performance battery; T1, tertile 1; T2, tertile 2; T3, tertile 3; TUG, timed up-and-go; WC, waist circumference; zCST, standardized chair-stand test; zHGS, standardized hand grip strength; Zn, zinc; zSMI, standardized skeletal muscle mass index; zSMM, standardized skeletal muscle mass.

### Stage 5: Collate, summarize, and report the results

Extracted data were collated into an Excel spreadsheet (Microsoft) and summarized using thematic analysis. The following 2 themes were identified: (1) the relationship of Zn intake (dietary with or without supplement) with nutritional status, sarcopenia, and frailty; and (2) the relationships of serum, hair, or nail Zn concentrations with the aforementioned factors.

## RESULTS

### Search results

**Figure 1:**
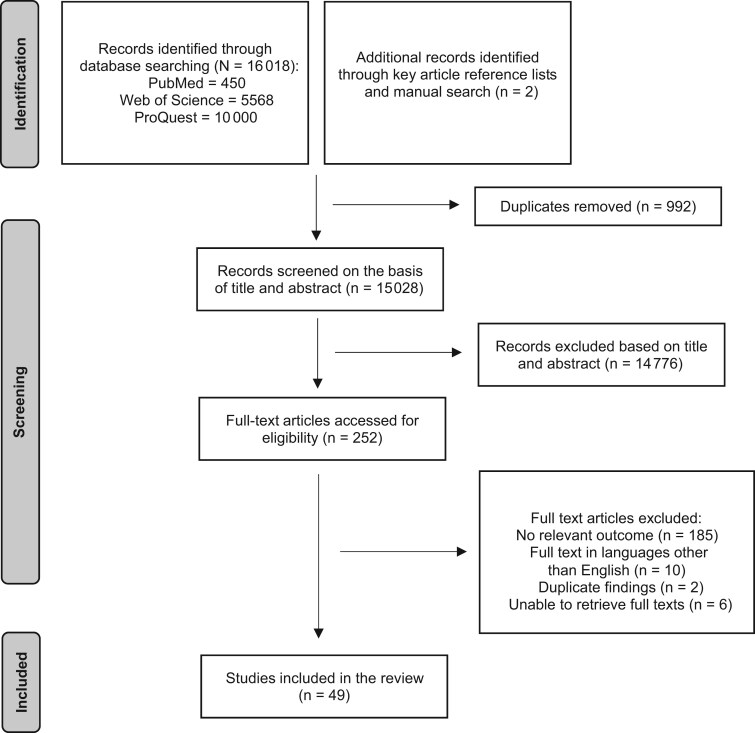
Preferred Reporting Items for Systematic Reviews and Meta-Analyses (PRISMA) flow diagram of the selection and inclusion of studies in this scoping review.

The original search of the 2 peer-reviewed databases (PubMed and Web of Science) conducted in August 2022 resulted in 6018 potentially relevant articles. In addition, 10 000 records extracted from the ProQuest database (the maximum number of records displayed by the database to avoid duplications) and sorted according to their relevance were also included. Manual searching identified 2 articles. Duplicate articles (n = 992) were removed using Endnote software, and the titles and abstracts of 15 028 studies were screened for eligibility. This resulted in 252 studies for full-text review using the inclusion and exclusion criteria. After excluding 203 articles, a total of 49 articles (including the 2 articles identified by the manual search) were included in the scoping review (Figure 1). Those articles were then categorized as those in which Zn intake was assessed (n = 30),^16–29^^,^^31–46^ serum or tissue Zn levels (n = 18),^47–64^ or both (n = 1).^30^ Two articles included different findings from the same study sample^54^^,^^64^; these findings were reported in combination in this review (Table 3).

**Table 3:** Characteristics and main findings of studies that reported relationships of tissue Zn concentration with nutritional status, sarcopenia, and frailty

Reference	Study area	Study design and sample	Assessment of Zn status and nutritional status	Primary findings related to the review question
Asaoka et al (2020)^47^	Tokyo, Japan	A hospital-based, retrospective cross-sectional study Study sample: consecutive outpatients ≥65 y old at a geriatric medical center N = 313	1. Serum Zn (μg/dL) 2. Frailty assessed by J-CHS criteria^a^	Serum Zn concentration was associated with frailty. Univariate association: mean (SD) serum Zn levels in nonfrail group: 74.3 (11.9); frail group: 69.6 (9.6); *P* = 0.002 Multivariate logistic regression analysis: standardized coefficient: –0.042, OR, 95%CI: 0.96 (0.92–0.99); *P* = 0.027
Bonaccorsi et al (2013)^48^	Florence, Italy	Cross-sectional study Study sample: Residents >64 y old of 12 nursing homes N = 428	1. Serum Zn (μg/L) 2. Nutritional status by BMI	No association between serum Zn and BMI was observed. Serum Zn by BMI categories, mean (SD): <19: M (n = 10): 863.00 (210.24); F (n = 36): 803.30 (152.05) >19 and <21: M (n = 14): 820.00 (146.66); F (n = 44): 814.50(141.07) >21 and <23: M (n = 11): 856.40 (137.13); F (n = 51): 816.50 (140.28) >23: M (n = 66), 850.50 (144.88); F (n = 196), 820.50 (136.70); for M and F, *P* > 0.05
Cheong et al (2020)^49^	Singapore	Cross-sectional study Study sample: Community-dwelling older adults aged ≥65 y N = 400 Serum Zn values were available only for 332 participants	1. Serum Zn (μg/L) 2. Nutritional status: BW, BMI, MUAC, CC, FM and MM by BIA	Serum Zn concentration was associated with BMI. Mean (SE) BMI in low-Zn group^a^ (n = 34; 25.61 ± 0.51) was higher than that of normal/high Zn group^a^ (n = 298; 24.30 ± 0.17; *P* = 0.0164). By multiple linear regression: estimate: –5.6; SE = 2.6; *P* = 0.0343 No associations between other parameters and Zn concentration were observed. 1. BW: low Zn, 64.8 (1.76); normal/high Zn, 61.3 (0.54), *P* = 0.0398 2. MUAC: low Zn, 28.74 (0.63); normal/high Zn, 27.58 (0.19), *P* = 0.0519 3. FM: low Zn, 19.06 (1.26); normal/high Zn, 17.58 (0.35), *P* = 0.1892 4. Fat %: low Zn, 29.12 (1.79); normal/high, 28.33 (0.50), *P* = 0.6212 5. MM: low Zn, 43.3 (1.73); normal/high Zn, 41.36 (0.47), *P* = 0.2007 6. CC: low Zn, 36.29 (0.42); normal/high Zn, 35.16 (0.19), *P* = 0.0481 Correlation between CC and serum Zn (Pearson *r =* –0.062; *P* = 0.2617) was not significant.
de Jong et al (2001)^50^	Dunedin, New Zealand	Cross-sectional study Study sample: Randomly selected community-dwelling women aged between 70 and 80 y N = 103, one outlier was excluded from serum Zn values	1. Serum Zn (μmol/L) 2. Physical functioning score^a^ by HGS, quadriceps strength, ADL, 6-m TUG	Serum Zn was associated with physical functioning score of older women. Mean serum Zn levels across tertiles of physical functioning score: low (n = 39), 12.0; medium (n = 31), 12.3; high (n = 33), 12.9; low vs high, *P* < 0.04
Gariballa et al (2020)^51^	South Yorkshire, United Kingdom	Hospital-based, prospective study with a follow-up at 6 wk and 6 mo Study sample: Acutely ill older patients aged ≥65 y Sample size at baseline: 432	1. Serum Zn (ng/mL) 2. Nutritional status by MM^a^	Serum Zn levels were significantly low in low-MM group at baseline and 6-wk follow-up, but not at 6-mo follow-up. Mean (SD) serum Zn levels in the 2 groups: 1. At baseline: low-MM group (n = 42): 571 (127); normal-MM group (n = 360): 629 (148); *P* < 0.05 2. At 6-w follow-up: low-MM group (n = 15): 605 (110); normal-MM group (n = 154): 684 (150); *P* < 0.05 2. At 6-mo follow-up: low-MM group (n = 9): 665 (150); normal-MM group (n = 151): 644 (138); *P* > 0.05
Gau et al (2020)^52^	Southeast Ohio, United States	Retrospective case-control study Study sample and size: community residents aged >50 y attending a geriatric clinic Case patients (n = 41): outpatients with Zn deficiency (serum Zn <0.66) Control group (n = 116): outpatients with normal Zn concentrations	1. Serum Zn (μg/mL) 2. Nutritional status by BMI	Serum Zn was not associated with BMI. Mean (SD) BMI of case patients, 28.6 (6.7); of control group: 27.9 (5.4); *P* = 0.479 Associations between BMI and Zn deficiency: adjusted OR, 1.07; 95%CI, 0.98–1.16; *P* = 0.136
Gau et al (2021)^53^	United States	Cross-sectional study using secondary data from 2 consecutive national surveys conducted in 2011–2012 and 2013–2014 Study sample: Participants aged >50 y N = 1136	1. Serum Zn (μg/mL) 2. Nutritional status by BMI	Serum Zn was associated with BMI. Number (%) of participants by BMI categories^a^ and serum Zn concentration tertiles^a^ were 1. Low Zn (n = 404): UW: 6 (1.49); normal: 108 (26.73); OW: 131 (32.43); obese: 159 (39.36) 2. Middle Zn (n = 380): UW: 10 (2.63); normal: 98 (25.79); OW: 136 (35.79); obese: 136 (35.79) 2. High Zn (n = 352): UW: 3 (0.85); normal: 95 (26.99); OW: 133 (37.78); obese: 121 (34.38); *P* = 0.031
Grieger et al (2007)^54^, (2009)^65^	Victoria, Australia	Cross-sectional study Study sample: Residents from an aging care facility N = 115	1. Serum Zn (μmol/L) 2. Nutritional status by TUG, MNA	Both TUG and MNA were associated with serum Zn. Negative correlation between TUG and serum Zn (n = 46; *r =* −0.449; *P* = 0.001). Mean TUG in low-serum Zn group was significantly low (*P =* 0.020). Low Zn^a^ (n = 19): 30.0 (3.3); adequate Zn^a^ (n = 27): 44.6 (5.6) Positive correlation between MNA score^a^ and serum Zn (n = 44; *r* = 0.307; *P* = 0.021)
Islam et al (2007)^55^	North Island of New Zealand	Cross-sectional study Study sample: Community residents aged ≥65 y N = 507	1. Hair and toenail Zn levels (μg/g) 2. Nutritional status by TUG	Hair and nail Zn concentrations were not associated with TUG. By multivariate logistic regression: hair Zn OR, 0.97, 95%CI, 0.79–1.20, *P* = 0.79; nail Zn OR, 0.83, 95%CI, 0.67–1.03, *P* = 0.09
Kvamme et al (2014)^56^	Tromsø, Norway	Cross-sectional study Study sample: Community-dwelling adults between 65 and 87 y old N = 1521	1. Serum Zn (μmol/L) 2. Nutritional status by MUST score^a^	Serum Zn was associated with malnutrition risk in men but not in women. Prevalence (no., %) of Zn deficiency^a^ in malnutrition categories: M: low risk, 84 (12); medium/high risk, 13 (31.7), *P* < 0.05 F: Low risk, 47 (6.7), medium/high risk, 10 (12.3), *P* = 0.07 Malnutrition risk according to the Zn status: M: normal Zn, 28 (4.3), Zn deficient, 13 (13.4), *P* = 0.01 F: normal Zn, 17 (9.8), Zn deficient, 10 (17.5), *P* = 0.07
Kosaka et al (2013)^30^	Japan	Cross-sectional study Study sample: Disabled elderly patients residing at nursing facilities N = 26	1. Serum Zn (μg/dL) 2. Nutritional status by BMI	Serum Zn concentration was associated with BMI. Mean (SD) serum Zn levels in BMI groups: low BMI (<18.5), 52 (6); normal BMI (≥18.5), 59 (8); *P* < 0.05
12. Lu et al (2021)^57^	China	Cross-sectional study Study sample: Community-dwelling older adults aged >60 y N = 3727	1. Serum Zn (μg/dL) 2. Nutritional status by BMI^a^	Serum Zn was associated with BMI. The median (IQR) serum Zn values were high in OW (103.0; 88.0–122.0) and obese (100.4; 86.2–116.9) older adults compared with those with UW (96.7; 80.4–120.0) and normal BMI (96.6; 82.0–116.6). χ^2^: 36.41, *P* < 0.001 Prevalence (%, 95%CI) of Zn deficiency^a^ was significantly low in OW and obese older adults (*P* < 0.001). UW (n = 252): 14.22 (9.63–18.81); normal (n = 1890): 10.32 (8.89–11.75); OW (n = 1147): 6.33 (4.91–7.76); obese (n = 438): 5.75 (3.43–8.07) Likelihood of Zn deficiency was low among OW and obese older adults. OR (95%CI) by multivariate logistic regression: UW: 1.33 (0.91–1.94), *P* = 0.14; normal: reference; OW: 0.67, 0.51–0.88, *P* < 0.01; obese: 0.52, 0.33–0.80, *P* < 0.01
Margetts et al (2003)^58^	United Kingdom	Secondary analysis of data from a national survey Study sample: Free-living and institutionalized older people ≥65 y old N = 1368	1. Serum Zn (μmol/L) Nutritional status: risk of undernutrition has assessed using specified criteria based on BMI and weight loss over past 6 mo^a^	Higher serum Zn levels were associated with lower risk of undernutrition. Mean (95%CI) serum Zn in the 3 risk groups: low risk (n = 1182): 14.1 (14.0–14.3); medium risk (n = 91): 13.2 (12.6–13.8); high risk (n = 95): 12.8 (12.2–13.4); high risk vs low risk, *P* < 0.05
Markiewicz-Zukowska et al (2015)^59^	Bialystok, Poland	Cross-sectional study Study sample: Residents of a nursing home Age range: 60–102 y N = 100	1. Serum Zn (mg/L) *2.* Nutritional status: BMI, fitness score calculated using FM and MM by BIA	Neither BMI nor fitness score was associated with serum Zn. Spearman correlation coefficients: BMI: *r* = –0.15, *P* > 0.05; fitness score: *r* = 0.19, *P* > 0.05
Mocchegiani et al (2012)^60^	Serente, Italy	Prospective cohort study Follow-up at 24 mo from baseline Study sample: community-dwelling adults aged ≥80 y Sample size: at baseline: 346; at 2-y follow-up: 221	1. Serum Zn *2.* Nutritional status: SPPB score by 4-m GS, CST, balance test, HGS	Serum Zn was associated with GS and SPPB score, but not with HGS or SPPB% decline^a^ at 2-yr follow up. Significant associations were lost when adjusted for inflammatory biomarkers. β (SE) in linear regression models: 1. Model 1 (adjusted for age, sex): GS: 0.032 (0.012), *P* = 0.01; SPPB: 0.425 (0.146), *P* < 0.01; HGS: 0.900 (0.505), *P* = 0.07; SPPB% decline: –2.633 (1.946), *P* = 0.18 2. Model 2 (adjusted by model 1 + BMI, HT, CHF, serum albumin/urea and cholesterol levels): GS: 0.026 (0.011), *P* = 0.02; SPPB: 0.352 (0.143), *P* = 0.01; HGS: 0.660 (0.497), *P* = 0.183; SPPB% decline: –2.256 (1.977), *P* = 0.254 3. Model 3 (adjusted by model 2 + CRP and IL-6): GS: 0.013 (0.012), *P* = 0.26; SPPB: 0.162 (0.145), *P* = 0.25; HGS: 0.153 (0.509), *P* = 0.76; SPPB% decline: –2.105 (2.097), *P* = 0.32
Peng et al (2010)^61^	Taiwan	Prospective study Follow-up every 3 mo for 12 Study sample: Severely disabled care-home residents N = 70	1. Serum Zn (μg/dL) 2. Nutritional status: BMI; triceps SFT, MAC	Serum Zn was associated with BMI and MAC, but not with SFT. Correlation coefficients (Spearman): BMI: –0.297, *P* = 0.013; SFT: 0.027, *P* = 0.822; MAC: –0.309, *P* = 0.009
Semba et al (2006)^62^	Baltimore, Maryland, United States	Prospective study Annual follow-up up to 36 mo Study sample: Community-dwelling women ≥65 y N = 766	1. Serum Zn (μg/mL) 2. Frailty assessed by modified Fried frailty phenotype criteria^a^	Serum Zn was associated with frailty status at the baseline. Serum Zn levels (geometric mean, 95%CI) in women with and without frailty at baseline: Frail (n = 246): 0.82 (0.80–0.85); nonfrail (n = 500): 0.87 (0.86–0.89); *P* = 0.001 Incidence of frailty over 3-yr interval was not associated with baseline serum Zn concentration. Incidence rates: lowest quartile of Zn at baseline (n = 101): 21; upper 3 quartiles of Zn at baseline (n = 347): 19.1; *P* = 0.52
Takeuchi et al (2018)^63^	Hyogo, Japan	Cross-sectional study Study sample: Community-dwelling women ≥65 y old N = 179	1. Serum Zn (μg/dL) 2. Nutritional status by HGS	Univariate association between serum Zn and HGS was observed, but significance was lost in multivariate model. Mean (SD) serum Zn levels: low HGS (<18), 74 (11); normal HGS, 78 (11); *P* = 0.007 By bivariate logistic regression: OR, 0.96, 95%CI, 0.93–0.99; *P* = 0.008
Xu et al (2022)^64^	Jiangsu, China	Case-control study Case group: Patients with sarcopenia Control group: Patients without sarcopenia Study sample: Older patients with chronic diseases admitted to a geriatric ward N = 121 (57 case patients; 64 control participants)	Serum Zn (umol/L) Sarcopenia was diagnosed using AWGS criteria^a^	Zn was a weak predictor of sarcopenia. Serum Zn levels were low in the sarcopenic group (*P* = 0.026). Serum Zn was associated with sarcopenic-related variables. SMI: *r* = 0.256, *P* < 0.01 HGS: *r* = 0.364, *P* < 0.01

a Detailed definitions are available in Table S2 in the Supporting Information online.

*Abbreviations:* ADL, activities of daily living; AWGS, Asian working group for sarcopenia; BIA, bioelectrical impedance analysis; BMI, body mass index; BW, body weight; CC, calf circumference; CHF, congestive heart failure; CI, confidence interval; CRP, C-reactive protein; CST, chair stand test; F, female; FM, fat mass; GS, gait speed; HGS, hand grip strength; HT, hypertension; IL-6, interleukin-6; IQR, interquartile range; J-CHS, the Japanese version of the Cardiovascular Health Study; M, male; MAC, mid-arm circumference; MM, muscle mass; MNA, mini nutritional assessment; MUAC, mid-upper arm circumference; MUST, malnutrition universal screening tool; OR, odds ratio; OW, overweight; SD, standard deviation; SE, standard error; SMI, skeletal muscle index; SFT, skinfold thickness; SPPB, short physical performance battery; TUG, timed up and go; UW, underweight; Zn, zinc.

### Study characteristics

The study characteristics are summarized in Tables 2 and 3 and Table S2 in the Supporting Information online.^16–64^

#### Years and regions

A majority of the studies (88%)^16–29^^,^^32–49^^,^^51–53^^,^^56^^,^^57^^,^^59–61^^,^^63^^,^^64^ were published after 2010, with the earliest publication being in 2001.^50^ The studies were conducted primarily in Europe (n = 18),^17^^,^^18^^,^^22^^,^^26^^,^^28^^,^^29^^,^^32^^,^^33^^,^^41–43^^,^^46^^,^^48^^,^^51^^,^^56^^,^^58–60^ 15 were conducted in Asia.^23^^,^^24^^,^^27^^,^^30^^,^^35^^,^^36^^,^^38^^,^^39^^,^^45^^,^^47^^,^^49^^,^^57^^,^^61^^,^^63^^,^^64^ Seven studies each were from North America^19^^,^^25^^,^^31^^,^^44^^,^^52^^,^^53^^,^^62^ and Australasia,^20^^,^^21^^,^^37^^,^^40^^,^^50^^,^^54^^,^^55^ and the remaining 2 studies were from Jordan^16^ and Turkey.^34^

#### Study designs and data types

Most research designs (n = 32)^17^^,^^19^^,^^21–31^^,^^33–36^^,^^38–41^^,^^46–50^^,^^54–57^^,^^59^^,^^63^ were cross-sectional, 9 studies were longitudinal,^18^^,^^20^^,^^32^^,^^37^^,^^42^^,^^51^^,^^60–62^ and follow-up ranged from 8 months^18^ to up to approximately 9 years.^42^ Four studies used a case-control research design.^16^^,^^43^^,^^52^^,^^64^ All of these studies were observational. The studies by Asaoka et al^47^ and Gau et al^52^ were based on retrospective data, and 4 articles were based on secondary analysis of data from large-scale surveys.^44^^,^^45^^,^^53^^,^^58^

#### Populations and samples

In most studies, the target populations were community-dwelling older adults (n = 33).^17^^,^^18^^,^^20–25^^,^^27^^,^^29^^,^^31–33^^,^^35–40^^,^^42–46^^,^^49^^,^^50^^,^^53^^,^^55–57^^,^^60^^,^^62^^,^^63^Institutionalized older adults participated in 9 studies.^16^^,^^19^^,^^28^^,^^30^^,^^34^^,^^48^^,^^54^^,^^59^^,^^61^ Three studies used both community-dwelling and institutionalized older adults as their study population.^26^^,^^41^^,^^58^ Three studies included outpatients of geriatric clinics or acutely ill patients who were admitted to a hospital.^47^^,^^51^^,^^52^ One study was based on patients with chronic diseases who were admitted to a geriatric ward,^64^ and in 2 studies,^30^^,^^61^ all the older adults were moderately or severely disabled.

Most studies included both male and female older adults (n = 42)^16–19^^,^^22^^,^^23^^,^^25–28^^,^^30–49^^,^^51–61^^,^^64^; 7 were restricted to 1 sex.^20^^,^^21^^,^^24^^,^^29^^,^^50^^,^^62^^,^^63^ The definition of the older population differed among studies: participants were ≥50 years in 5 studies,^17^^,^^23^^,^^37^^,^^52^^,^^53^ ≥60 years in 11 studies,^16^^,^^18^^,^^25^^,^^26^^,^^29^^,^^35^^,^^38^^,^^42^^,^^44^^,^^57^^,^^59^ ≥65 years in 23 studies,^19^^,^^22^^,^^24^^,^^27^^,^^28^^,^^31–34^^,^^39^^,^^41^^,^^43^^,^^45^^,^^47–49^^,^^51^^,^^54–56^^,^^58^^,^^62^^,^^63^ and ≥70 years in 7 studies.^20^^,^^21^^,^^30^^,^^36^^,^^40^^,^^50^^,^^60^ Three studies did not clearly mention the age cutoffs they used.^46^^,^^61^^,^^64^

Sample size ranged from 26^30^ to 3727,^57^ with 44 studies (89%) having a sample size >100.^17–27^^,^^29^^,^^31–45^^,^^47–60^^,^^62–64^ Specific exclusion criteria, in any, applied to the studies, and population characteristics are summarized in Table S2 in the Supporting Information online.

#### Assessment of Zn status

Among the studies that measured Zn intake, most studies used 24-hour dietary recalls to collect dietary intake data (n = 11),^16–18^^,^^25^^,^^28^^,^^31^^,^^34^^,^^36^^,^^38^^,^^40^^,^^45^ and food frequency questionnaires,^22–24^^,^^29^^,^^32^^,^^33^^,^^37^^,^^41^ diet history,^20^^,^^21^^,^^27^^,^^35^^,^^39^^,^^42^ and 3-day diet records^26^^,^^43^^,^^44^^,^^46^ were used by 8, 6, and 4 studies, respectively. Zn intake was calculated using weighted food intake^19^ and meal menu charts^30^ by 1 study each. All studies that assessed Zn concentrations reported serum Zn levels^30^^,^^47–54^^,^^56–64^ except for 1 that used hair and toenail samples.^55^

#### Assessment of nutritional status, sarcopenia, and frailty

Tables 2 and 3 briefly outline the definitions used for nutritional risk, physical function status, BMI and body composition categories, and sarcopenia and frailty diagnoses in selected studies. Detailed definitions are summarized in Table S2 in the Supporting Information online. Among the 9 studies that reported findings related to sarcopenia,^16^^,^^21^^,^^23^^,^^35^^,^^36^^,^^41^^,^^43^^,^^47^^,^^64^ 4 and 3 studies used the Asian Working Group for Sarcopenia^35^^,^^36^^,^^47^^,^^64^ and European Working Group on Sarcopenia in Older People (EWGSOP)^21^^,^^23^^,^^41^ criteria, respectively. One study used both EWGSOP and the Foundation of the National Health Institutes of Health criteria.^21^ No information about the sarcopenia screening method was mentioned in 1 study,^16^ and in 1 study criteria, BMI was considered.^43^

Twelve studies screened for frailty using different criteria.^20^^,^^22^^,^^24^^,^^27^^,^^33^^,^^38–40^^,^^42^^,^^45^^,^^47^^,^^62^ The majority of the studies used the Fried frailty phenotype criteria,^20^^,^^22^^,^^27^^,^^38^^,^^40^^,^^42^^,^^45^^,^^62^ and 3 studies used the Japanese version of the Cardiovascular Health Study criteria.^24^^,^^39^^,^^47^ Malnutrition risk has been assessed using BMI and weight loss,^58^ Mini Nutritional Assessment,^26^^,^^34^^,^^65^ and the Malnutrition Universal Screening Tool.^56^

The studies included in this review used different methods to assess body composition and components of sarcopenia and frailty, such as bioelectrical impedance analysis, dual-energy X-ray absorptiometry, skinfold thickness, hand grip strength, chair stand test, gait speed (GS) or walking speed for different distances, balance tests, and anthropometric measurements.

### Relationship of Zn intake with nutritional status, sarcopenia, and frailty

Our review summarizes evidence from 31 studies that assessed the relationship of Zn intake with nutritional status, sarcopenia, or frailty (Table 2). One study reported that Zn intake in older women was negatively associated with both BMI and waist circumference.^31^ Another study reported a significant positive association between appendicular lean mass and Zn intake at baseline and over 2.6 years.^37^ Relationships between anthropometric parameters (calf circumference, skeletal MM, and BMI) reported in 5 other studies were nonsignificant.^19^^,^^25^^,^^29^^,^^30^^,^^46^ Two studies revealed that Zn intake was significantly lower among older adults who are malnourished or at risk of malnutrition, using Mini Nutritional Assessment as the screening tool, than those with normal nutritional status.^26^^,^^34^

Eight studies assessed the relationship between Zn intake and sarcopenia,^16^^,^^21^^,^^23^^,^^28^^,^^35^^,^^36^^,^^41^^,^^43^ whereas only 2 studies reported that higher Zn intake was associated with a lower risk of sarcopenia.^21^^,^^36^ Many studies assessed the relationships between Zn intake and different sarcopenia-related variables, including HGS,^18^^,^^32^^,^^46^ lower extremity strength,^37^^,^^46^ GS,^32^^,^^44^ and physical fitness.^17^^,^^18^^,^^33^^,^^42^ Among those, 1 study revealed a significant positive association between Zn intake and Short Physical Performance Battery (SPPB) score.^42^ The study further showed that a higher intake of Zn and a lower intake of foods rich in phytate pointed to a reduced risk of impaired lower extremity function. Another study reported that older adults with slow GS were more likely to be in the lowest quartile of Zn intake.^44^

The association between Zn intake and frailty was reported in 9 studies.^20^^,^^22^^,^^24^^,^^27^^,^^38–40^^,^^42^^,^^45^ Kaimoto et al^27^ revealed significantly lower Zn intake in older women with prefrailty, whereas contrasting findings were reported by Hayashi et al.^24^ The study by Vega-Cabello et al^42^ concluded that the risk of frailty is inversely associated with Zn intake, its bioavailability, and a lower intake of phytate-rich foods. Furthermore, they found that older women who adhered to the recommended Zn intake (by the Institute of Medicine) had a lower risk of frailty. Significant associations between Zn intake and frailty were reported in 4 other studies,^22^^,^^38^^,^^39^^,^^45^ whereas 2 studies found that associations are significant only in men.^39^^,^^45^ Another study also reported a higher prevalence of inadequate Zn intake among prefrail older men.^40^ The other study did not find significant associations between Zn intake and incident prefrailty or frailty.^20^

### Relationship of Zn status with nutritional status, sarcopenia, and frailty

Table 3 summarizes the findings, from 19 studies, related to the relationship of Zn concentrations with nutritional status, sarcopenia, or frailty. Of these studies, 10 provided data on associations between anthropometric measurements and serum Zn concentrations.^30^^,^  ^48^^,^^49^^,^^52^^,^^53^^,^^57–59^^,^^61^^,^^64^Three studies reported positive associations^30^^,^^53^^,^^57^and three reported no^48^^,^^52^^,^^59^associations between BMI and serum Zn concentrations, whereas 2 studies reported positive associations.^49^^,^^61^ Another study reported an inverse association between serum Zn concentrations and risk of undernutrition assessed with BMI and weight loss over 6 months.^58^ Data related to the relationship of serum Zn concentration with calf circumference, mid-upper arm circumference, fat mass, MM, and triceps skinfold thickness were also reported in 4 studies.^49^^,^^51^^,^^61^^,^^64^ Among those, Peng et al^61^ reported an inverse association with mid-upper arm circumference, whereas Gariballa et al^51^ reported a positive association with the MM at baseline and 6-week follow-up. The prevalence of Zn deficiency was significantly higher in older men who were at medium and high risk of malnutrition per the Malnutrition Universal Screening Tool criteria.^56^ Another study reported a positive correlation of serum Zn with Mini Nutritional Assessment score.^65^

Xu et al^64^ reported significant associations between serum Zn concentrations and sarcopenia and sarcopenia-related variables, skeletal muscle index, and HGS. Among the 2 studies that assessed timed up-and-go, 1 reported a negative association with serum Zn.^54^ In contrast, the other revealed no association with hair and nail Zn concentrations.^55^ Another study assessed the physical performance of older women and reported a positive association.^50^ Takeuchi et al^63^ found significant associations between serum Zn and low HGS among older women in bivariate analysis; however, the association was lost in multivariate analysis. Mocchegiani et al^60^ found no associations between serum Zn and SPPB, GS, and HGS. Evidence of inverse associations between frailty and serum Zn concentrations was included in 2 studies.^47^^,^^62^

## DISCUSSION

Zn deficiency, undernutrition, sarcopenia, and frailty are commonly found among the older population. A recent report, based on the discussions of the European Society for Clinical and Economic Aspects of Osteoporosis, Osteoarthritis and Musculoskeletal Diseases working group (8 September 2016), has stated that it is important to have “healthier” dietary patterns in older age with adequate intakes of protein, long-chain polyunsaturated fatty acids, vitamin D, and antioxidant nutrients^66^; however, little was mentioned about the role of Zn in prevention and management of frailty and sarcopenia. Therefore, in this review, we went beyond the prevalence of these nutritional problems, aiming to explore their relationship. The information collated in this scoping review highlights the gaps in the current literature and provides the necessary basis for future studies focused on the potential use of Zn in the prevention and management of malnutrition, sarcopenia, and frailty in older adults.

Studies addressing the associations between Zn status and individual anthropometric and sarcopenia-related variables, including BMI, waist circumference, calf circumference, MM, fat mass, GS, and HGS, have shown inconsistent results. Antioxidant nutrients have been suggested to prevent the loss of MM and muscle function by decreasing muscle fiber oxidation and inactivating the reactive oxygen species. As such, antioxidant-rich diets are proposed to be important in ameliorating sarcopenia progression. However, the existing evidence shows inconsistent associations among Zn status, sarcopenia, and muscle parameters. A recent meta-analysis conducted using 4 studies did not observe significant differences in Zn intake between older adults with and without sarcopenia (pooled standardized mean difference, −0.60 mg; 95%CI, −1.31 to 0.12 mg; *I*^2^ = 0%).^67^ Besides variations in the measurement methods and equipment used (eg, MM measured by skinfold thickness, bioelectrical impedance analysis, or dual-energy X-ray absorptiometry), inconsistencies may lie in the methodological differences of the studies. On the other hand, strong associations were found between Zn status and malnutrition or risk of malnutrition assessed using screening tools such as Mini Nutritional Assessment^26^^,^^34^^,^^65^ and Malnutrition Universal Screening Tool,^56^ suggesting the consideration of multiple factors associated with the nutritional status rather than focusing on 1 measurement.

Our review shows positive and negative associations, as well as no associations, between serum Zn concentrations and BMI. Several explanations have been proposed for the underlying mechanisms for serum Zn levels and overweight or obesity. One theory suggests that obesity-associated chronic inflammation and stress promote Zn absorption by adipocytes as a result of enhanced expression of metallothionein and Zn transporters.^68^ Conversely, Zn deficiency can induce the release of reactive oxygen species and cause oxidative stress in tissues, leading to obesity-related complications. Low dietary intake of Zn in older individuals can make them highly vulnerable to oxidative stress and inflammation, resulting in sarcopenic obesity. Another theory proposes that low serum Zn concentrations impair the leptin-signaling pathway and elevated levels of circulating leptin, causing leptin resistance. In addition, increased Zn excretion was observed in obesity.^69^ On the other hand, the positive associations observed may be because overweight and obese individuals are more likely to consume Zn-rich foods such as meats, seafood, and nuts than people of normal and low weight.^57^

Acquired Zn deficiency is a potentially undiagnosed disorder, and the only clearly demonstrated sign among older adults is impaired immune defense, whereas other signs, such as impaired taste and wound healing, are less frequently observed.^70^ Serum Zn concentrations can be affected by various factors, including dietary intake; the status of Zn-binding proteins such as albumin, transferrin, and α-2-macroglobulin, diabetes mellitus, gastrointestinal disorders leading to malabsorption, heavy alcohol use, medications and various medical conditions that result in Zn depletion, and foods high in phytate content.^70^^,^^71^ Noteworthy, the study by Vega-Cabello et al^42^ included in this review reported that older adults with lower intake of high-phytate foods have a reduced risk of impaired lower extremity function and frailty compared with those with high intake even if their dietary Zn intake is similar. Nevertheless, available evidence also indicates that there is no clear relationship between serum Zn concentrations and Zn intake.^72^^,^^73^ According to the estimations of a recent dose-response meta-analysis, only a 6% change in plasma or serum Zn concentrations was resulted by doubling the Zn intake.^74^

The recent report on European Society for Clinical Nutrition and Metabolism micronutrient guidelines states that it is essential to interpret plasma Zn concentrations together with changes in serum albumin levels and the effect of inflammation.^70^ Plasma Zn concentration decreased with the magnitude of the inflammatory response; therefore, to make a reliable clinical interpretation, the C-reactive protein level should be <20 mg/L.^75^ Moreover, circulating zinc negatively correlates with interleukin-6, interleukin-8, and tumor necrosis factor-α levels.^70^ However, few studies included in this review took these factors into consideration. The study conducted by Mocchegiani et al^60^ reported that significant associations observed between serum Zn level, GS, and SPPB in the unadjusted model and the model adjusted for serum albumin were lost when adjusted for inflammatory biomarkers (C-reactive protein and interleukin-6).

The studies included in this review showed that older adults with sarcopenia had poor Zn status related to loss of MM and function. Zn is an essential micronutrient for MM synthesis; thus, the presence of Zn deficiency can cause a reduction in MM, leading to physical and functional impairment.^69^ Zn deficiency may further promote sarcopenia by interfering with antioxidant responses and autophagy. The characteristic features of frailty include low physical activity levels, slow motor performance, and weakness, which may be caused by the decline in MM and strength. Many studies that assessed the relationship between frailty and Zn status reported inverse associations,^27^^,^^40^^,^^42^^,^^47^^,^^62^ despite the diversity of study designs, population characteristics, and frailty diagnostic criteria. The importance of antioxidants in frailty has been reported in previous studies. An inverse association was reported by Kobayashi et al^76^ between frailty and dietary total antioxidant capacity (assessed by oxygen radical absorbance capacity, ferric-reducing ability of plasma, Trolox [a vitamin E analog] equivalent antioxidant capacity, and total radical-trapping antioxidant parameter) among older Japanese women.^76^

The prevention of sarcopenia and frailty remains a major challenge in medicine and public health, requiring urgent attention. Furthermore, the prevention of micronutrient and antioxidant deficiencies has been suggested as a potential intervention to reduce the risk of frailty and sarcopenia. The role of Zn in the prevention of sarcopenia and frailty is yet to be determined due to the conflicting findings reported in the studies included in this review. Alternative approaches to analyzing the Zn status, such as the ratio of serum Cu to Zn levels (CZr), has used in some studies. Gaier et al^77^ found significant correlations between CZr and MM, muscle strength and power, lower extremity function, and SPPB decline. However, individual Cu and Zn levels were not correlated with decline in physical function. Another study has also concluded that CZr is a more reliable parameter than serum Cu and Zn concentrations to discriminate patients with physical disabilities from healthy persons.^78^

Furthermore, some studies have assessed associations between sarcopenia and dietary and nutrient patterns rather than evaluating individual dietary intake. The better dietary pattern with a mean (SD) Zn intake of 6.97 (1.52) mg was a significant predictor of sarcopenia (Odds ratio = 2.790; 95%CI, 1.394–5.58; *P =* 0.004) than the poorer dietary pattern in which the mean Zn intake was 4.84 (1.14) mg among long-term-care residents.^79^ In contrast, a nutrient pattern that was high in vitamins and Zn, among others, did not show a significant association with sarcopenia.^80^ Many studies have revealed the associations between self-reported physical activity, activities of daily living (ADL), and Zn status. One study reported that those who met the recommended exercise intensity and frequency for older adults had lower Zn intake (Male—11.1 mg, Female—8.4 mg) than those who did not (Male—13.6 mg, Female—9.2 mg).^81^ Another study reported significantly lower serum Zn levels in physically disabled older adults (67.2 μg/dL) than healthy (128.1 μg/dL) controls and a positive correlation between serum Zn and activities of daily living score (*r* = 0.449; *P* < 0.01).^78^ In contrast, studies that assessed the associations of serum Zn with ADL^82^^,^^83^ and instrumental activities of daily living did not report significant findings.^83^ Davis et al^84^ reported a low Zn intake in older adults with high perceived physical fatigability.

Strengths of this scoping review include the comprehensive search of 3 large and reliable databases (PubMed, Web of Science, ProQuest) without using limitations in publication years and study design. In combination with the manual search we conducted, it is possible that all the pertinent articles were identified. However, there are limitations to this scoping review. First, we did not evaluate the quality of evidence included in the review, which is an inherent feature of scoping reviews. Second, the findings cannot be generalized, because of the differences in study samples, designs, diagnostic criteria, and assessment methods used in the studies. The diversity of results also limited the extent of the analysis. Third, we included articles that were published only in English. Finally, associations, rather than causation, have been reported in studies. Nevertheless, future studies should focus on longitudinal, prospective aspects to investigate the potential relationships between Zn status and sarcopenia and frailty.

Studies that explored the differences in the above relationships with population characteristics (eg, male and female, community-dwelling and institutionalized elderly) are scarce, suggesting the need for such future studies. In addition, we noted gaps in pertinent literature from low- and middle-income countries, although these countries are experiencing rapid population aging. A major reason for this might be the lack of infrastructure and financial support to enable comprehensive studies in this context. Only 1 study included in this review assessed both Zn intake and concentrations, making it difficult to compare Zn screening methods. However, associations of sarcopenia and frailty were more evident for serum Zn concentrations than Zn intake. Zn and nutritional status are complex phenomena with myriad physical and social influences. Therefore, the use of comprehensive and validated tools and criteria to screen and assess the nutritional status of older adults is recommended because individual measures are not reliable in isolation. Moreover, using international consensus definitions in sarcopenia and frailty screening will delineate the associations.

## CONCLUSIONS

In this scoping review, we found inconsistent results in the relationship of Zn intake and tissue Zn levels with individual nutritional measurements. The associations were more evident for serum Zn levels with malnutrition, sarcopenia, and frailty. Sufficient data are missing to compare population characteristics and clarify causative relationships, indicating the need for well-designed prospective studies. There is a need for studies from low- and middle-income countries to fill the gaps in the current literature.

## Supplementary Material

nuad094_Supplementary_Data

## Supporting Information

The following Supporting Information is available through the online version of this article at the publisher’s website.

*Table S1*  **Preferred Reporting Items for Systematic Reviews and Meta-Analyses Extension for Scoping Reviews (PRISMA-ScR) checklist**

*Table S2*  **Exclusion criteria, population characteristics, and definitions used in selected studies**

*File S1* **PubMed search strategy**
