# Supplementary material for: The role of zinc on nutritional status, sarcopenia, and frailty in older adults: a scoping review
Source: Nutr Rev. 2023 Aug 7;82(7):988–1011. doi: 10.1093/nutrit/nuad094 (PMC11163457; doi:10.1093/nutrit/nuad094)
Supplement: nuad094_Supplementary_Data [file nuad094_supplementary_data.zip › nuad094_Supplementary_Data/Supplementary file 1.docx]

**PubMed search strategy**

("Elderly"[Title/Abstract] OR "older people"[Title/Abstract] OR "older person*"[Title/Abstract] OR "senior*"[Title/Abstract] OR "ageing"[Title/Abstract] OR "Nonagenarian"[Title/Abstract] OR "Octogenarian"[Title/Abstract] OR "Centenarian"[Title/Abstract] OR "aged"[MeSH Terms] OR "aging"[MeSH Terms] OR "frail elderly"[MeSH Terms] OR "aged, 80 and over"[MeSH Terms]) AND ("zinc"[Title/Abstract] OR "dietary zinc"[Text Word] OR "zinc intake"[Text Word] OR "plasma zinc"[Text Word] OR "serum zinc"[Text Word] OR "zinc/deficiency"[MeSH Terms] OR "zinc/blood"[MeSH Terms] OR "trace elements"[MeSH Terms]) AND ("nutritional status"[Title/Abstract] OR "muscle mass"[Title/Abstract] OR "lean body mass"[Title/Abstract] OR "fat free mass"[Title/Abstract] OR "body muscle*"[Title/Abstract] OR "muscle waste"[Title/Abstract] OR "muscle loss"[Title/Abstract] OR "strength"[Title/Abstract] OR "muscle strength"[Title/Abstract] OR "dynapenia"[Title/Abstract] OR "strength loss"[Title/Abstract] OR "physical performance"[Title/Abstract] OR "physical function*"[Title/Abstract] OR "functional mobility"[Title/Abstract] OR "muscles"[MeSH Terms] OR "muscle, skeletal"[MeSH Terms] OR "muscular atrophy"[MeSH Terms] OR "muscle strength"[MeSH Terms] OR ("paresis"[MeSH Terms] OR "muscle weakness"[MeSH Terms]) OR "mobility limitation"[MeSH Terms] OR "physical fitness"[MeSH Terms] OR "sarcopenia"[MeSH Terms] OR "Frailty"[MeSH Terms])

No. of records extracted: 450
