# Supplementary material for: The role of zinc on nutritional status, sarcopenia, and frailty in older adults: a scoping review
Source: Nutr Rev. 2023 Aug 7;82(7):988–1011. doi: 10.1093/nutrit/nuad094 (PMC11163457; doi:10.1093/nutrit/nuad094)
Supplement: nuad094_Supplementary_Data [file nuad094_supplementary_data.zip › nuad094_Supplementary_Data/Supplementary Table 2-2023.07.07.docx]

Supplementary Table 2: Exclusion criteria, population characteristics and definitions used in selected studies

| **Author and year** | **Exclusion criteria** | **Population characteristics (age, sex, etc.)** | **Definitions used** |
| --- | --- | --- | --- |
| 1. Al-Majali et al. (2021)^1^ | Those with muscle-wasting diseases like cancer, T1DM, PEM, disability, and Alzheimer's disease | Age, mean (SD):  Sarcopenic Group - 77.5 (6.9);  Non-sarcopenic group - 74.7 (5.5)  Sex, M: 12 (48%) |  |
| 2. Aparicio-Ugarriza  et al. (2019)^2^ | 1. Those who are institutionalized  2. Those suffering from a physical or mental illness that would have affected PF tests or questionnaires responses | Sex, M: 130 (40%)  Age, mean (SD):  M - 64.9 (6.5); F - 67.2 (6.7)  *Number of participants in PF levels;*  Low PF: M - 30, F - 37  Medium PF: M - 59, F - 95  High PF: M - 41, F - 62 | Score for each PF test (CST, 8-foot TUG, 6-min walk test, HGS) ranged from 0 (worst) to 3 (best) points, resulting the maximum score of 12 points.  *Different levels of PF;*  Low PF: 0-3 points  Medium PF: 4-8 points  High PF: 9-12 points |
| 3. Asamane et al. (2020)^3^ | Those who are institutionalized or suffering from dementia or any mental illness | Age, Mean (SD):  Baseline - 70.8 (8.1); Follow-up - 70.7 (8.2)  Sex, M (%):  Baseline - 59%; Follow-up - 62%  SPPB score, median (IQR):  Baseline - 11.1 (4.0); Follow-up - 10.0 (4.0)  HGS, mean (SD):  Baseline - 27.6 (9.8); Follow-up - 26.5 (9.5) |  |
| 4. Carrier et al. (2021)^4^ | 1. Those who are receiving palliative care, are medically unstable (hospital admission/s in previous month), requiring tube feeding or end-of-life care  2. Those who recently or temperarily admitted to care homes | Age, mean (SD): 86.8 (7.8)  Sex, M (%): 31.2  CC, mean (SD): 33.3 (4.8)  Low CC, %: 32.6  Zn NAR score, mean (SD): 0.81 (0.16) | - |
| 5. Das et al. (2020)^5^ | Those living in a residential aged care facility | Age, Mean (SD): 81.4 (4.61)  Frailty status at baseline (n=767), n (%);  Robust - 341(44.5)  Pre-frail - 362 (47.2)  Frail - 64 (8.3)  Frailty status at 3-year follow-up (n=330), n (%):  Robust - 134 (40.6)  Pre-frailty - 175 (53)  Frailty - 21 (6.4)  Number of participants in quartiles of Zn intake at baseline:  Q1 - 76, Q2 – 88, Q3 - 85, Q4 - 81 | Frailty was diagnosed using Fried Fraility phenotype criteria:  Weight loss, Reduced muscle strength by HGS, Exhaustion, Low PA, Slow GS (6-m)  *Frailty categories;*  Pre-frail: met one or two of the above criteria  Frail: met 3 or more of the above criteria  Quartiles of Zn intake:  Q1: <11.00  Q2: 11.01-13.31  Q3: 13.32-16.74  Q4: >16.75 |
| 6. Das et al. (2021)^6^ | - | Age, Mean (SD):  81.1 (4.5)  Prevalence of sarcopenia by different criteria, n (%):  1. FNIH - 89 (12.9)  2. EWGSOP - 84 (12.9)  3. EWGSOP2 - 93 (13.8)  Probable sarcopenia - 208 (31%)  Severe sarcopenia - 21 (3.1)  Proportion of participants met the NRV for Zn - 65.8%  No. of subjects in each Zn intake quartile:  Q4 - 81, Q3 - 85, Q2 - 88, Q1 - 76 | Sarcopenia was diagnosed using three definitions.  1. EWGSOP criteria:  ALM/height^2^ <7.25 with HGS <30  and/ or GS<0.8  2. EWGSOP2 criteria:  ALM/height^2^ <7.0, HGS <27, GS<0.8  3. FNIH criteria:  ALM/BMI<0.789 and HGS<26kg  Zn intake quartiles:  Q1: <11, Q2: 11.01-13.31,  Q3: 13.32-16.74, Q4: >16.75 |
| 7. De Nucci et al. (2022)^7^ | - | Age, mean (SD): 73.4 (6.3)  Sex, M: 749 (53.5%)  Prevalence of frailty, n (%): 204 (13.6) | Frailty was diagnosed using Fried Fraility phenotype criteria:  Weight loss, Reduced muscle strength (CST>15), Exhaustion, Low PA, Slow 5-m GS (<0.6)  Frail: met 3 or more of the above criteria |
| 8. Ebrahimi-Mousavi et al. (2022)^8^ | 1. Those with limited mobility, history of debilitating diseases like active cancer and organ failure.  2. Those who could not walk without crutches, walkers or assistive devices, or had artificial limbs or prostheses. | Age, mean: 66.8  Sex, M: 150 (50)  Prevalence of sarcopenia, n (%): 54 (18) | Sarcopenia was diagnosed using EWGSOP criteria:  1. ALM by DEXA/ Height^2^ <5.45 for women and <7.26 for men and  2. Low HGS - using age and gender specific cut-off values or  3. 4-m GS <0.8 |
| 9. Hayashi et al. (2021)^9^ | 1. Those who require long-term nursing care.  2. Those who were institutionalized.  3. Those who were diagnosed with dementia  4. Those who could perform ADL independently  5. Those who have received dietary restriction instructions | Age, Median (IQR): 73 (68 – 76)  Prevalence of prefrailty, n (%): 54 (45)  Zn intake, median (IQR): 8.5 (7.9 – 9.1)  Proportion of participants with inadequate Zn intake, n (%): 9 (7.5) | Frailty was diagnosed using J-CHS criteria:  1. Weight loss >2kg over 6 months  2. Low PA  3. Exhaustion  4. HGS <18  5. 6-m GS <1  Prefrailty: Presence of 1-2 deficits  Non-frailty: No deficits  Inadequate Zn intake:  65-74 years old: <7 mg  >75 years old: <6mg |
| 10. Jun et al. (2020)^10^ |  | Sex, M: 1462 (49.2%) | Weight categories:  1.HW: BMI 18·5–24·9  2.OW: BMI 25–29·9  3.Obese: BMI ≥ 30 |
| 11. Jyväkorpi et al. (2016)^11^ |  | Mean age (SD) and % of female sex in each MNA class:  Normal - 85 (6.8), 75  At risk - 82.4 (6.8), 73  Malnutrition - 78.5 (7.4), 55 | MNA classes:  12-14: normal nutritional status  8 – 11: at risk of malnutrition  0-7: malnourished |
| 12. Kaimoto et al. (2021)^12^ | Those with history of diagnosis of dementia | Age, mean:  Total - 74.9, M - 74.5, F - 51.9  Sex, M: 300 (36.8%)  Prevalence of prefrailty, n (%): 432 (53) | Frailty was diagnosed using Fried Fraility phenotype criteria:  Exhaustion, Slow GS, Muscle weakness (low HGS), Low PA, Weight loss in the past 6 months  1. Robust: met 0 criteria  2. Pre-frail: met >1 |
| 13. Keser et al. (2021)^13^ | 1. Those with cancer related cachexia  2. Those with contraindication for BIA such as pacemaker or amputation  3. Those who cannot sit independently with or without help. | Sex, M: 15 (17.8%)  Age, mean (SD):  M - 86.3 (3.5); F - 80.7 (6.8)  *Body composition, mean (SD):*  1. MM (% BW):  M - 14.9 (2.1); F - 21.8 (2.1)  2. FM (% BW):  M - 42.2 (5.9); F - 36.7 (5.4)  *Zn intake, mean (SD):*  M - 6.5 (2.9); F - 5.6 (2.6) | *Body composition categories:*  *Osteo-sarcopenic adiposity:*  Simultaneous presence of ;  1. sarcopenia (MM with the S-score*< -0.1)  2. osteopenia (Total bone mass with T-score* <-1.0) and,  3. adiposity (Total FM of >25% for men and >32% for women) |
| 14. Khanal et al. (2021)^14^ | 1. Those with mobility-related symptoms  2. Those with a history of muscle or nervous system conditions | Age, mean (SD): 70 (7)  SMM, mean (SD) – 16.9 (3.1)  SMM_r_, mean (SD): 25.8 (4.0) | Relative SMM (SMM_r_): (SMM/ body mass) x 100  SMM_r_ categories:  1. Low SMM_r_ <22.1%  2. High SMM_r_ >22.1% |
| 15. Kosaka et al. (2013)^15^ |  | Age, Mean (SD): 90 (6)  Sex, M: 6 (23%)  BMI, mean (SD): 19.4 (3.2) | - |
| 16. Ledikwe et al. (2003)^16^ | Those with Poor congitive function and depression | Sex, M: 81 (45.3%)  Age, mean (SD):  M - 73.3(5.0); F - 73.5(5.0)  BMI and WC, mean (SD):  M - 28.5 (3.6), 103.7 (9.6)  F - 28.3 (4.7), 89.1 (11.4)  Zn intake, mean (SD):  M - 10.1 (3.6); F - 7.1 (5.2) |  |
| 17. Lengele et al. (2020)^17^ | - | Sex, M: 93 (39.1%)  Age, median (IQR): Baseline -72 (70-78)  Follow-up - 76 (73-81)  *GS, median (IQR):*  Baseline – 1.2 (1.0-1.3); Follow-up – 1.2 (1.0-1.4)  *HGS, median (IQR):*  Baseline  M - 39.5 (34.5-44.0); F - 21.0 (18.0-24.0)  Follow-up  M - 32.0 (26.5-39.0); F - 16.0 (12.0-18.5)  *Zn intake, median (IQR):*  Baseline - 6.6 (5.9-7.4); Follow-up - 6.4 (5.6-7.2) |  |
| 18. Moradell et al. (2021)^18^ | 1. Those suffering from dementia and cancer.  2. Those with the SPPB score <4. | Age, mean (SD): 80.4 (6.0)  Sex, M: 23 (22.8%) | SPPB categories:  Frail: 4-6 points  Pre-frail: 7-9 points  Robust: >9 points |
| 19. Ongan et al. (2015)^19^ | 1. Those who suffering from psychiatric disorders, dementia  2. Those who are living in the institution for <3 months. | Age, mean (SD): 76.1 (7.3)  Sex, n (%):  M - 58 (64.6); F - 196 (35.4) | *MNA categories:*  1. Normal: score >23.5  2. At risk: score 23.5 -17.0  3. Malnourished: score <17 |
| 20. Otsuka et al. (2021)^20^ | - | Age, mean (SD): 71.2 (7.4)  Sex, M: 437 (32.5%)  Prevalence of sarcopenia, n (%): 77 (5.7) | Sarcopenia was diagnosed referring to AWGS criteria:  1. Age >60 or >65 years  2. ALM by BIA/ height^2^ <7 for men and <5.7 for women  3. HGS <26 in men and <18 in women  4. GS <0.8  Sarcopenia: presence of 1 and 2 with 3 or 4. |
| 21. Park et al. (2022)^21^ | Not defined. | Sex, M: 385 (48%)  Prevalence, n (%): sarcopenia - 111 (13.9)  Severe sarcopenia - 10 (1.25)  Age, mean (SE):  Non-sarcopenic - 76 (0.2), Sarcopenic - 78.2 (0.4)  ALM, mean (SE):  Non-sarcopenic - 6.56 (0.04), Sarcopenic - 5.65 (0.07)  HGS, mean (SE):  Non-sarcopenic - 24.9 (0.3), Sarcopenic - 19.0 (0.4)  GS, mean (SE):  Non-sarcopenic - 0.94 (0.01), Sarcopenic - 1.06 (0.03) | Sarcopenia was diagnosed referring to AWGS criteria:  1. Decreased MM: ALM by DEXA/Height <7 for men and <5.4 for women  2. Low muscle strength: HGS <26 for men and <18 for women  3. Low physical performance:  GS (m/s) <0.8  Sarcopenia – Presence of both 1 and 2  Severe sarcopenia:  Presence of all 1,2 and 3 |
| 22. Scott et al. (2010)^22^ | - | Age, mean (SD):  Baseline - 61.9 (7.1) Follow-up - 64.6 (7.1)  Sex, F: 50.1%  ALM, mean (SD):  Baseline - 24.6 (5.3); Follow-up - 23.3 (4.9)  Leg strength, mean (SD):  Baseline - 30.8 (11.1); Follow-up - 32.2 (10.9) | - |
| 23. Shalini et al. (2020)^23^ | - | Age, median (IQR): 65 (62 – 67)  Sex, M: 89 (55%)  Prevalence of frailty, n (%): 33 (20)  Zn intake, median (IQR): 6.8 (5.9 – 8.1) | Frailty was diagnosed using modified Fried Fraility phenotype criteria:  1. Unintentional weight loss of >4.5kg in the previous year  2. Exhaustion  3. Low PA  4. Low HGS and 5-m GS: specified cut-off values by gender and BMI  Non-frail: met 0 to 2 criteria, Frail: met >3 criteria |
| 24. Tamaki et al. (2018)^24^ | Those with decreased cognitive function | Age, mean (SD): 72.6 (5.8)  Sex, M: 254 (31.8)  Prevalence of frailty:  By KCL criteria - 8.4%  J-CHS criteria - 4.0% | Frailty was diagnosed using two criteria:  1. Kihon checklist (KCL) criteria -  Questionnaire consists of 8 domains.  8-25 points: Frail  4-7 points: Pre-frail  0-3 points: Robust  2. J-CHS criteria  - Weight loss  - Exhaustion  - Low PA  - Low HGS (26kg for men and <18kg for women)  -Low GS (<1m/s)  Robust: met none of the criteria  Pre-frail: met 1 or 2 criteria  Frail: met >3 criteria |
| 25. Tay et al. (2021)^25^ | - | Age, median (IQR): 80 (77 - 84)  Sex:  M - 191 (41.1%), F - 274 (58.9)  Zn intake (mg), median (IQR):  M - 8.56 (6.60 - 10.49)  F - 6.85 (5.35 - 8.79) | Frailty was diagnosed using modified Fried Fraility phenotype criteria:  1. Weight loss of >5% in the prior year  2. Weak HGS <30 for men and <20 for women  3. Low 3-m GS <0.8  4. Exhaustion  5. Low PA  Presence of 1 to 2 in the above |
| 26. ter Borg et al. (2016)^26^ | 1. Those with a poor cognitive function (MMSE score<24)  2. Those who are unable to perform assessments (prosthesis, pacemaker, wheelchair bound or bedridden, severe active rheumatoid arthritis, acute angina pectoris,  poststroke status with evident lingering symptoms, diseases of the nervous system, or dementia) | Age, median (IQR): 74 (69 - 79)  Sex, M: 110 (49%)  Prevalence of sarcopenia, n (%): 53 (23)  SMM, median (IQR): 23.5 (17.1-28.4)  SMI, median (IQR): 8.3 (6.7-9.5)  HGS, mean (SD): 26.4 (9.7)  GS, mean (SD): 1.01 (0.27) | SMI (kg/m^2^): SMM by BIA/height^2^  Sarcopenia was diagnosed using EWGS criteria:  1. Low MM:  SMI <10.75 for men, <6.75 for women  2. Low muscle strength:  HGS <30 for men and <20 for women  3. Low GS (4-m) <0.8 |
| 27. Vega-Cabello et al. (2022)^27^ | - | Sex, M: 1379 (46.5)  Tertiles of Zn intake (number of participants, median):  T1 - 987, 7.79  T2 - 988, 8.91  T3 - 988, 10.40  Age across Zn intake tertiles, mean (SD):  T1 - 69.5 (6.7)  T2 - 69 (6.4)  T3 - 68.6 (6.4)  Incidence rates of;  Impaired LEF - 515  Frailty - 241 | *Impaired LEF:*  Each component of SPPB scored on a 4-point scale, and the total score ranges from 0 to 12.  Impaired LEF <6 points in SPPB score  Frailty was diagnosed using modified Fried Fraility phenotype criteria:  1. Unintentional weight loss >4.5 kg in the previous year  2. Exhaustion  3. Low PA  4. Slow walking speed (lowest quantile of GS adjusted for sex and height)  5. Muscle weakness by HGS in the dominant hand (lowest quantile of HGS adjusted for sex and BMI)  Frailty – met 3 or more of the above conditions |
| 28. Verlan et al. (2017)^28^ | - | Age, mean (SD): 71 (4)  Sex, M: 27 (40.9%) | SMI (%): (SMM by DEXA/BW) x100  Sarcopenia diagnosis:  - SPPB score of 4-9  - Low SMI (<37% for men and <28% for women)  - BMI between 20-30  Controls (non-sarcopenic):  - SPPB score of 11-12  - Normal SMI (<1SD below the sex-specific mean for young adults using BIA or DEXA)  - BMI between 20-30 |
| 29. Waters et al. (2014)^29^ | - | Sex, M: 121 (38.4)  Subjects with slow GS by sex, n (%):  M - 27 (22.3); F - 57 (29.4)  Age of subjects in each GS category by sex, mean:  M: Slow GS - 79.1, Normal GS - 76.2  F: Slow GS - 81.1, Normal GS - 74.3 | - |
| 30. Wu et al. (2022)^30^ | 1. Those with dementia, mental dysfunction, or schizophrenia  2. Those who were under a therapeutic diet | Age, mean: 74  Number of participants in frailty groups by age and sex:  *M, 65-74 years*  Robust – 231; Pre-frail – 156; Frail – 13  *M, >74 years*  Robust – 86; Pre-frail – 117; Frail – 26  *F, 65-74 years*  Robust – 224; Pre-frail – 127; Frail – 10  *F, >74 years*  Robust – 60; Pre-frail – 109; Frail – 27 | Frailty was diagnosed using modified Fried Fraility phenotype criteria:  1. Unintentional weight loss in the previous year  2. Self-reported exhaustion  3. Low PA  4. Low HGS and GS (4-m) screened using specified cut-off values by gender and BMI  Robust: met none of the criteria  Pre-frail: Met 1 or 2 criteria  Frail: >3 criteria |
| 31. Yeung et al. (2021)^31^ | No exclusion criteria applied | Age, mean (SD): 77.2 (9.0)  Sex, n (%):  M - 20 (34.5); F - 38 (65.5)  Mean (SD) of nutritional parameters:  1. SMM (n=49): 24.4 (5.8)  2. SMI (n=49): 9.26 (1.34)  3. SMM/BMI (n=49): 0.88 (0.20)  4. HGS (n=45): 22.7 (8.3)  CST (n=57), median (IQR): 21.3 (16.3-38.7)  Energy -adjusted Zn intake (mg), median (IQR): 8.4 (7.6-9.9) | SMI (kg/m^2^): SMM/height^2^ |
| 32. Asaoka et al. (2020)^32^ | 1. Patients with severe cardiac, pulmonary, musculoskeletal, neurological disorders, delirium tremens, gastrectomy, IBD, malignant diseases, type-1DM, hypo/hyper-thyroidism, hypo/hyper-parathyroid disorders, mental illness  2. Those with a history of acute cerebrovascular, GI, renal, coronary, hepatic, or respiratory events  3. Immobile patients | Age, mean (SD): 75.7 (6.0)  Sex, M: 134 (42.8%)  Prevalence of frailty, n (%): 71 (22.7)  Serum Zn, mean (SD): 73.2 (11.5) | Frailty was diagnosed using J-CHS criteria:   1. Unintentional weight loss of >2kg within 6 months  2. Self-reported fatigue  3. Self-reported physical inactivity  4. Low HGS (M<26, F<18)  5. Low GS (<0.8) |
| 33. Bonaccorsi et al. (2013)^33^ | Those with terminal illnesses | Age, mean (SD): M - 81.14 (7.89) F - 84.41 (7.61)  Sex, M: 101 (23.6%)  BMI, Mean (SD):  M - 24.78 (4.80); F - 25.48 (5.79)  Serum Zn, Mean (SD):  M - 848.10 (149.80); F - 817.20 (139.04) | - |
| 34. Cheong et al. (2020)^34^ | 1. Those with following conditions within the last 30 days from the screening visit (dementia, type 1 or 2 DM, any active infectious disease, severe GI disorders, cystic fibrosis, end-stage organ or pre-terminal diseases, and acute myocardial infarction)  2. Those with an active malignancy within 5 years of the data collection  3. Those with difficulties in ambulation, oral intake, communication and following instructions  4. Those with MUST score >0 at screening | Age, Mean (SE): 71.21 (0.26)  Sex, M: 183 (45.75%)  Serum Zn values, n: M - 181, F - 215  Mean (SE) of nutritional parameters:  *BW:* 61.86 (0.48)  *BMI:* 24.53 (0.15)  *MUAC:* 27.73 (0.16)  *CC:* 35.24 (0.16)  *FM:* 17.87 (0.32)  *Fat%:* 28.50 (0.45)  *MM:* 41.57 (0.41)  Serum Zn, mean (SE): 862.4 (7.0) | Serum Zn categories:  Low: <724  Normal: 724 – 1244  High: >1244 |
| 35. de Jong et al. (2001)^35^ | 1. Institutionalized  2. non-ambulatory  3. Terminal illness | Age, Mean (SD) : 74.9 (3.1)  Serum Zn, mean (SD) : 12.4 (1.4) | Physical functioning score:  Sum of HGS and Quadriceps strength, ADL, and 6-m TUG |
| 36. Gariballa et al. (2020)^36^ | 1. Those with severe medical or psychiatric illness including malignancy and dementia  2. Those who are living in an institution | Age, range: 65-92 years  Sex, M: 221(51%) | MAMC = MAC – (3.14 x Triceps SFT)  Low MM:  MAMC <21.1 cm for men and <19.2 cm for women |
| 37. Gau et al. (2020)^37^ | Those with severe renal insufficiency, serum Zn concentration >1.2μg/ml, or hospitalization within 2 months prior to data collection | Age, Mean (SD):  Cases - 82.6 (8.7)  Control - 78.2 (8.9)  Sex, M:  Cases - 12 (29%), Controls - 43 (37%) | - |
| 38. Gau et al. (2021)^38^ | - | Age in serum Zn tertiles, mean (SE):  Low - 67.3 (0.5)  Middle - 66.9 (0.7)  High - 66.1 (0.4)  Sex according to Zn tertiles, n:  *Low:* M - 203, F- 201  *Middle:* M - 172, F - 208  *High:* M - 186, F - 166  n (%) of participants in BMI categories;  UW - 19 (1.67)  Normal - 301 (26.50)  OW - 400 (35.21)  Obese - 416 (36.62) | *Serum Zn terciles:*  Low: 0.409-0.748  Middle: 0.749-0.870  High: 0.872-1.698  *BMI categories:*  UW – BMI <18.5  Normal - BMI 18.5-24.9  OW - BMI 25.0-29.9  Obese - BMI>30 |
| 39. Grieger et al. (2007),^39^  (2009)^40^ | 1. Those who are residents in rehabilitation, palliative care, and dementia wards  2. Those who are frail/ bed bound/ poor cognition | Age, mean (SD): 80.2 (10.6)  Sex, M: 37 (32%)  TUG time, Mean (SD): 36 (21)  Serum Zn, Mean (SD): 11.2 (2.8) | Serum Zn categories:  Low Zn <10.7  Adequate >10.7  Adequate MNA screening score: >12 |
| 40. Islam et al. (2007)^41^ | - | Age, mean (SD): 65 (11)  Sex, M: 269 (56%)  TUG, Mean (SD):  M - 10.6 (6.69); F - 10.4 (7.84)  Hair Zn, Mean (SD):  M - 138.9 (35.09); F - 148.4 (43.87)  Nail Zn, Mean (SD):  M - 103.6 (32.57); F - 105.40 (52.51) | - |
| 41. Kvamme et al. (2014)^42^ | - | Age, mean (SD): 71.9 (5.6)  Sex, M: 743 (48.9%)  BMI, mean (SD): 26.9 (4.1)  Serum Zn, mean (SD): 11.9 (1.8)  Prevalence, n (%):  Zn deficiency  Total - 154 (10.1), M - 97 (13.1), F - 57 (7.3)  Malnutrition:  Total - 122 (8.0), M - 41 (5.5), F - 81 (10.4) | Risk of malnutrition has assessed by Modified MUST.  1. Weight loss over the last 6 months  2. BMI  MUST score;  0- low risk  1 – medium risk  >2 – High  *Zn deficiency:*  M: <10.7 (AM) and 9.3 (PM)  F: <10.1 (AM) and 9.0 (PM) |
| 42. Lu et al.  (2021)^43^ |  | Age, mean (SD): 71.8 (7.5)  Sex, M: 1894 (50.8%)  Serum Zn, Median (IQR): 99.2 (84.3-118.7)  Prevalence of Zn deficiency, % (95% CI): 8.68 (7.74-9.61)  BMI, mean (SD): 23.6 (3.6) | *BMI categories:*  UW: <18.5  Normal: 18.5 - <24  OW: 24 - <28  Obese: >28  Zn deficiency: serum Zn <74 for men and <70 for women |
| 43. Margetts et al. (2003)^44^ | - | Sex, M: 694 (50.7%)  Participants in each age group, n (%):  65-74: 507 (37.1)  75-84: 527 (38.5)  >85: 334 (24.4) | Risk of undernutrition;  1. High: BMI<18.5 or BMI 18.5-20 with weight loss >3.2kg  or BMI>20 with weight loss >6.4kg  2. Medium: BMI 18.5-20 with weight loss <3.2kg  or BMI>20 with weight loss 3.2-6.4kg  3. Low: BMI>20 and no weight loss |
| 44. Markiewicz-Zukowska et al. (2015)^45^ | - | Sex, M: 48  Age, mean (SD): 76 (11)  BMI, mean (SD) : 27.5 (6.5)  Serum Zn, mean (SD): 0.83 (0.20) | - |
| 45. Mocchegiani et al. (2012)^46^ |  | Sex, M: 115 (33.2%)  Age, mean (SD): M - 86.08 (4.85) F - 85.73 (4.84)  SPPB decline in 2-years, mean (SD): 1.41 (0.19)  SPPB% decline in 2-years, mean (SD): 18.13 (2.5) | SPPB% decline:  (Baseline SPPB – Follow-up SPPB) x 100/Baseline SPPB |
| 46. Peng et al. (2010)^47^ |  | Age, mean (SD): 77.7 (8.2)  Sex, M: 64.3%  BMI at baseline, mean (SD): 20.2 (3.8)  SFT at baseline, mean (SD): 10.8 (5.7)  MAC at baseline, mean (SD): 25.3 (3.5)  Serum Zn, mean (SD): 86.8 (20.2) |  |
| 47. Semba et al. (2006)^48^ |  | Fraility status at baseline:  Frail - 250  Not frail - 516  Age, mean (SD):  Frail - 80.4 (7.9),  Not frail - 76.1 (7.4) | Frailty was diagnosed using modified Fried Fraility phenotype criteria:  i) self-reported unintentional weight loss of 10 pounds or more in the prior year  ii)self-reported exhaustion  iii) Weakness (HGS adjusted by BMI)  iv) Slow GS (adjusted by height)  v) low PA |
| 48. Takeuchi et al. (2018)^49^ | Those who were under treatment for acute or chronic inflammatory diseases, cancer, cardiovascular, hepatic and renal diseases. | Prevalence of low HGS, n (%): 68  Age, mean (SD):  Low HGS - 81.1 (5.5)  Normal HGS - 74.2 (8.7) |  |
| 49. Xu et al. (2022)^50^ | - | Age, Mean (95% CI):  Cases - 76 (70-81)  Controls - 67 (59-71)  Sex, M:  Cases - 34 (59.6%),  Controls - 23 (35.9%) | SMI: ALM by DEXA/height^2^  Sarcopenia was diagnosed using AWGS criteria:  I. SMI<7 for men and <5.4 for women  II. HGS <28 for men and <18 for women  III. 6-m GS <1 and 5-time CST >12  If I and either II or III criterion met, it indicated sarcopenia. |

Abbreviations: ALM, appendicular lean mass; AWGS, Asian working group for sarcopenia; BIA, bioelectrical impedance analysis; BMI, body mass index; BW, body weight; CC, calf circumference; CST, chair stand test; DEXA, dual-energy X-ray absorptiometry; EWGSOP, European working group on sarcopenia in older people; F, female; FM, fat mass; FNIH, Foundation of the National Health Institutes of Health; GS, gait speed; HGS, hand grip strength; HW, healthy weight; IQR, inter-quartile range; J-CHS, Japanese version of the cardiovascular health study; KCL, Kihon checklist; LEF, lower extremity function; M, male; MAC, mid-arm circumference; MAMC, mid-arm muscle circumference; MM, muscle mass; MMSE, mini mental state examination; MNA, mini nutritional assessment; MUAC, mid-upper arm circumference; MUST, malnutrition universal screening tool; NAR, nutrient adequacy ratio; NRV, nutrient reference values; OW, overweight; PA, physical activity; PEM, protein energy malnutrition; PF, physical fitness; SD, standard deviation; SE, standard error; SFT, skinfold thickness; SMI, skeletal muscle index; SMM, skeletal muscle mass; SPPB, short physical performance battery; T1DM, type 1 diabetes Mellitus; TUG, timed up-and-go; UW, underweight; WC, waist circumference; Zn, Zinc.

*S-score/ T-score – standard deviation of SMM/ total bone mass with respect to healthy references individuals between 25 to 30 years old

**References**

1. Al-Majali SZ, Ghazzawi HA, Amawi AT. Evaluation of Nutrients Intake for a Group of Jordanian Older Adults with Sarcopenia Syndrome in Amman: An Explorative and Pilot Study. J Aging Res. 2021;6641967.
2. Aparicio-Ugarriza R, Luzardo-Socorro R, Palacios G, et al. What is the relationship between physical fitness level and macro- and micronutrient intake in Spanish older adults?. Eur J Nut. 2019;58(4):1579–1590.
3. Asamane EA, Greig CA, Thompson JL. The association between nutrient intake, nutritional status and physical function of community-dwelling ethnically diverse older adults. BMC Nutr. 2020;6:36.
4. Carrier N, Villalon L, Lengyel C, et al. Diet quality is associated with malnutrition and low calf circumference in Canadian long-term care residents. BMC Nut. 2019;5 (57).
5. Das A, Cumming RG, Naganathan V, et al. Prospective Associations Between Dietary Antioxidant Intake and Frailty in Older Australian Men: The Concord Health and Ageing in Men Project. J Gerontol A Bio Sci. 2020;75(2):348–356.
6. Das A, Cumming RG, Naganathan V, et al. Associations between nutrient intakes and dietary patterns with different sarcopenia definitions in older Australian men: the concord health and ageing in men project. Public Health Nutr. 2021;24(14):4490–4505.
7. De Nucci S, Zupo R, Donghia R, et al. Dietary profiling of physical frailty in older age phenotypes using a machine learning approach: the Salus in Apulia Study. Eur J Nut. 2022. ]
8. Ebrahimi-Mousavi S, Hashemi R, Bagheri A, Heshmat R, Dorosty-Motlagh A, Esmaillzadeh A. Association between dietary intake of branched-chain amino acids and sarcopenia and its components: a cross-sectional study. Sci Rep. 2022;12(1):5666.
9. Hayashi T, Fukuda Y, Sato R, Ogasawara M, Tamura, K. Association of physical prefrailty with prevalence of inadequate nutrient intake in community-dwelling Japanese elderly women: A cross-sectional study. Asia Pac J Clin Nutr. 2021;30(2):263–274.
10. Jun S, Cowan AE, Bhadra A, et al. Older adults with obesity have higher risks of some micronutrient inadequacies and lower overall dietary quality compared to peers with a healthy weight, National Health and Nutrition Examination Surveys (NHANES), 2011-2014. Public Health Nutr. 2020;23(13):2268–2279.
11. Jyväkorpi SK, Pitkälä KH, Puranen TM, et al. High proportions of older people with normal nutritional status have poor protein intake and low diet quality. Arch Gerontol Geriatr. 2016;67:40–45.
12. Kaimoto K, Yamashita M, Suzuki T, et al. Association of Protein and Magnesium Intake with Prevalence of Prefrailty and Frailty in Community-Dwelling Older Japanese Women. J Nutr Sci Vitaminol. 2021;67(1):39–47.
13. Keser I, Cvijetic S, Ilic A, Baric IC, Boschiero D, Ilich JZ. Assessment of Body Composition and Dietary Intake in Nursing-Home Residents: Could Lessons Learned from the COVID-19 Pandemic Be Used to Prevent Future Casualties in Older Individuals?. Nutrients. 2021;13(5):1510.
14. Khanal P, He LX, Degens H, et al. Dietary Protein Requirement Threshold and Micronutrients Profile in Healthy Older Women Based on Relative Skeletal Muscle Mass. Nutrients. 2021;13(9):3076.
15. Kosaka K, Yamashita S, Ando C, Endo Y, Taniguchi K, Kikunaga S. Relationships among Body Mass Index, Activities of Daily Living and Zinc Nutritional Status in Disabled Elderly Patients in Nursing Facilities. J Nutr Sci Vitaminol. 2013;59(5):420–430.
16. Ledikwe JH, Smiciklas-Wright H, Mitchell DC, Jensen GL, Friedmann JM, Still CD. Nutritional risk assessment and obesity in rural older adults: a sex difference. Am J Clin Nutr. 2003;77(3):551–558.
17. Lengele L, Moehlinger P, Bruyere O, Locquet M, Reginster JY, Beaudart C. Association between Changes in Nutrient Intake and Changes in Muscle Strength and Physical Performance in the SarcoPhAge Cohort. Nutrients. 2020;12(11):3485.
18. Moradell A, Fernández-García ÁI, Navarrete-Villanueva D, et al. Functional Frailty, Dietary Intake, and Risk of Malnutrition. Are Nutrients Involved in Muscle Synthesis the Key for Frailty Prevention? Nutrients. 2021;13(4):1231.
19. Ongan D, Rakıcıoğlu N. Nutritional status and dietary intake of institutionalized elderly in Turkey: a cross-sectional, multi-center, country representative study. Arch Gerontol Geriatr. 2015;61(2):271-6.
20. Otsuka Y, Iidaka T, Horii C, et al. Dietary Intake of Vitamin E and Fats Associated with Sarcopenia in Community-Dwelling Older Japanese People: A Cross-Sectional Study from the Fifth Survey of the ROAD Study. Nutrients. 2021;13(5):1730.
21. Park SJ, Park J, Won CW, Lee HJ. The Inverse Association of Sarcopenia and Protein-Source Food and Vegetable Intakes in the Korean Elderly: The Korean Frailty and Aging Cohort Study. Nutrients. 2022;14(7):1375.
22. Scott D, Blizzard L, Fell J, Giles G, Jones G. Associations Between Dietary Nutrient Intake and Muscle Mass and Strength in Community-Dwelling Older Adults: The Tasmanian Older Adult Cohort Study. J Am Geriatr Soc. 2010,58(11),2129–2134.
23. Shalini T, Chitra PS, Kumar BN, Madhavi G, Reddy GB. Frailty and Nutritional Status among Urban Older Adults in South India. J Aging Res. 2020;8763413.
24. Tamaki K, Kusunoki H, Tsuji S, et al. The Relationship between Dietary Habits and Frailty in Rural Japanese Community-Dwelling Older Adults: Cross-Sectional Observation Study Using a Brief Self-Administered Dietary History Questionnaire. Nutrients. 2018;10(12):1982.
25. Tay E, Barnett D, Leilua E, et al. The Diet Quality and Nutrition Inadequacy of Pre-Frail Older Adults in New Zealand. Nutrients. 2021;13(7):2384.
26. ter Borg S, de Groot LCPGM, Mijnarends DM, et al. Differences in Nutrient Intake and Biochemical Nutrient Status Between Sarcopenic and Nonsarcopenic Older Adults—Results From the Maastricht Sarcopenia Study. J Am Med Dir Assoc. 2016;17(5):393–401.
27. Vega-Cabello V, Caballero FF, Lana A, et al. Association of Zinc Intake With Risk of Impaired Physical Function and Frailty Among Older Adults. J Gerontol A Biol Sci. 2022;77(10):2015–2022.
28. Verlaan S, Aspray TJ, Bauer JM, et al. Nutritional status, body composition, and quality of life in community dwelling sarcopenic and non-sarcopenic older adults: A case-control study. Clin Nutr. 2017;36(1):267–274.
29. Waters DL, Wayne SJ, Andrieu S, et al. Sexually dimorphic patterns of nutritional intake and eating behaviors in community-dwelling older adults with normal and slow gait speed. J Nutr Health Aging. 2014;18(3):228–233.
30. Wu S-Y, Lee S-C, Yeh N-H, et al. Dietary Characteristics of Elders with Frailty and with Mild Cognitive Impairment: Cross-Sectional Findings and Implications from the Nutrition and Health Survey in Taiwan 2014-2017. Nutrients. 2022;14(24):5216.
31. Yeung SSY, Reijnierse EM, Deen P, Trappenburg MC, Meskers CGM, Maier AB. Nutrient Intake and Muscle Measures in Geriatric Outpatients. J Am Coll Nutr. 2021;40(7):589–597.
32. Asaoka D, Takeda T, Inami Y, et al. The Association between Frailty and Abdominal Symptoms: A Hospital-based Cross-sectional Study. Intern Med. 2020;59(14):1677-1685.
33. Bonaccorsi G, Lorini C, Assad GB, Pepe P, Santomauro F. Serum trace elements and risk of malnutrition in institutionalised elderly. Eur J Clin Nutr. 2013;67(2):155–160.
34. Cheong M, Chew STH, Oliver J, et al. Nutritional Biomarkers and Associated Factors in Community-Dwelling Older Adults: Findings from the SHIELD Study. Nutrients. 2020;12(11):3329.
35. de Jong N, Gibson RS, Thomson CD, et al. Selenium and zinc status are suboptimal in a sample of older New Zealand women in a community-based study. J Nutr. 2001;131(10):2677–2684.
36. Gariballa S, Alessa A. Associations between low muscle mass, blood-borne nutritional status and mental health in older patients. BMC nutr. 2020;6:6.
37. Gau JT, Ebersbacher C, Kao TC. Serum Zinc Concentrations of Adults in an Outpatient Clinic and Risk Factors Associated With Zinc Deficiency. J Am Osteopath Assoc. 2020;120(11):796–805.
38. Gau JT, Chavan B, Li Y, Clark BC, Haile ZT. Association between serum zinc levels and basic physical functioning: secondary data analysis of NHANES 2011-14. BMC Nutr. 2021;7(1):57.
39. Grieger J, Nowson C, Ackland ML. Anthropometric and biochemical markers for nutritional risk among residents within an Australian residential care facility. Asia Pac J Clin Nutr. 2007;16(1):178–186.
40. Grieger JA, Nowson CA, Ackland LM. Nutritional and functional status indicators in residents of a long-term care facility. J Nutr Elder. 2009;28(1):47–60.
41. Islam S, Carter K, Mhurchu CN, Anderson CS. Selenium and zinc in hair and toenails in relation to the physical and mental health status of older adults: The Freemasons Health study in New Zealand. Australas J Ageing. 2007;26(4):180-186.
42. Kvamme J-M, Grønli O, Jacobsen BK, Florholmen J. Risk of malnutrition and zinc deficiency in community-living elderly men and women: the Tromsø Study. Public Health Nutr. 2015;18(11):1907–1913.
43. Lu JX, Hu YC, Li M, et al. Zinc Nutritional Status and Risk Factors of Elderly in the China Adult Chronic Disease and Nutrition Surveillance 2015. Nutrients. 2021;13(9):3086.
44. Margetts BM, Thompson RL, Elia M, Jackson AA. Prevalence of risk of undernutrition is associated with poor health status in older people in the UK. Eur J Clin Nutr. 2003;57(1):69–74.
45. Markiewicz-Zukowska R, Gutowska A, Borawska MH. Serum Zinc Concentrations Correlate with Mental and Physical Status of Nursing Home Residents. Plos One. 2015;10(1):e0117257.
46. Mocchegiani E, Malavolta M, Lattanzio F, et al. Cu to Zn ratio, physical function, disability, and mortality risk in older elderly (ilSIRENTE study). Age. 2012;34(3):539–552.
47. Peng L-N, Liang C-K, Chou M-Y, et al. Association between serum copper, zinc and hospital admissions among care home residents. Arch Gerontol Geriatr. 2010;51(1):E24–E7.
48. Semba RD, Bartali B, Zhou J, Blaum C, Ko CW, Fried LP. Low serum micronutrient concentrations predict frailty among older women living in the community. J Gerontol A Biol Sci. 2006;61(6):594–599.
49. Takeuchi M, Tsuboi A, Minato S, et al. Elevated serum adiponectin and tumor necrosis factor-α and decreased transthyretin in Japanese elderly women with low grip strength and preserved muscle mass and insulin sensitivity. BMJ Open Diabetes Res Care. 2018;6(1):e000537.
50. Xu B, Guo Z, Jiang B, et al. Factors affecting sarcopenia in older patients with chronic diseases. Ann Palliat Med. 2022;11(3):972–983.
